# MELEGROS: Monolithic Elephant‐Inspired Gripper with Optical Sensors

**DOI:** 10.1002/advs.202518878

**Published:** 2026-02-02

**Authors:** Petr Trunin, Diana Cafiso, Anderson Brazil Nardin, Trevor Exley, Lucia Beccai

**Affiliations:** ^1^ Soft BioRobotics Perception Istituto Italiano di Tecnologia (IIT) Genova Italy; ^2^ The Open University, Affiliated Research Centre at Istituto Italiano di Tecnologia (ARC@IIT) Istituto Italiano di Tecnologia Genova Italy

**Keywords:** bioinspiration, monolithic, pneumatic actuation, simulation of architected structures, soft optical sensors, supportless 3D printing, triply periodic minimal surface

## Abstract

The elephant trunk exemplifies a natural gripper where structure, actuation, and sensing are seamlessly integrated. Inspired by the distal morphology of the African elephant trunk, we present MELEGROS, a Monolithic ELEphant‐inspired GRipper with Optical Sensors, emphasizing sensing as an intrinsic, co‐fabricated capability. Unlike multi‐material or tendon‐based approaches, MELEGROS directly integrates six optical waveguide sensors and five pneumatic chambers into a pneumatically actuated lattice structure (12.5 mm cell size) using a single soft resin and one continuous 3D print. This eliminates mechanical mismatches between sensors, actuators, and body, reducing model uncertainty and enabling simulation‐guided sensor design and placement. Only four iterations were required to achieve the final prototype, which features a continuous structure capable of elongation, compression, and bending while decoupling tactile and proprioceptive signals. MELEGROS (132 g) lifts more than twice its weight, performs bioinspired actions such as pinching, scooping, and reaching, and delicately grasps fragile items like grapes. The integrated optical sensors provide distinct responses to touch, bending, and chamber deformation, enabling multifunctional perception. MELEGROS demonstrates a new paradigm for soft robotics where fully embedded sensing and continuous structures inherently support versatile, bioinspired manipulation.

## Graphical abstract

MELEGROS is a monolithic, elephant‐inspired soft gripper integrating 3D‐printed optical sensors within a pneumatically actuated lattice. Simulation‐guided design decouples tactile and proprioceptive sensing. Characterized through grasping tests, MELEGROS can reach, scoop, and grasp, showcasing a paradigm where fully embedded sensing and continuous structure enable versatile, bioinspired soft robotic manipulation.

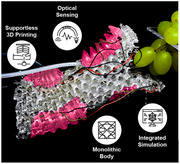

## 1 Introduction

The elephant trunk is a remarkably versatile biological manipulator that integrates sensing and actuation within a jointless structure to grasp objects of many shapes, weights, and sizes. In the African elephant, the trunk tip has two asymmetric finger‐like projections enabling pinching, scooping, and supporting actions [1, 2, 3]. The absence of any division between the continuum arm and the tip, combined with distributed sensory feedback, supports smooth reaching, grasping, and highly dexterous manipulation tasks (e.g., ripping leaves from a wrapped branch). Unlike engineered systems that separate sensing, actuation, and structure, the elephant trunk links them through the integration of muscles, connective tissue, skin, and embedded mechanoreceptors. This arrangement allows the elephant to control movement and respond efficiently to contact with its environment, even with occluded vision during prehensile activity [4]. *In this sense, the trunk is monolithic: structure, actuation, and sensing are inseparable*.

This type of integration is still uncommon in robotic systems. Although today robots can deform and adapt to their surroundings through compliant materials and structures [5, 6, 7], many are built by combining separate sensing, actuation, and structural elements [8, 9, 10]. This gap is related to a broader challenge in soft robotics: the lack of truly monolithic systems that combine all functional elements into a single material body. In particular, this problem stems from the fact that common transduction mechanisms, e.g., resistive [11, 12, 13, 14] and capacitive [15, 16, 17, 18] sensors, depend on conductive materials, with inherent mechanical characteristics (e.g., stiffness) different from the ones typically used for the robot's body. Moreover, sensing elements are often added post‐fabrication. This leads to mechanical mismatches that can compromise compliance and induce failure under cyclic loading. Recent efforts have achieved various degrees of actuator–sensor integration [19]. For example, Truby et al. combined EMB3D printing with molding to produce soft somatosensitive actuators by injecting conductive ionogel into elastomeric matrices [20], while Xiao et al. fabricated a fully 3D‐printed robotic hand incorporating soft capacitive sensors via dual‐extrusion of dielectric and conductive silicones [21]. In addition to transduction strategies, the pressure feedback from fluidic channels has been leveraged to maintain material uniformity [22], though at the expense of increased design complexity (thin hollow channels) and potential performance trade‐offs between sensing and actuation. Although these approaches represent meaningful advances toward the monolithic approach, they still rely on multiple materials, involve elaborate fabrication workflows, and restrict design versatility. To fully eliminate material mismatches and post‐assembly procedures, a solution is to build sensors with the same material as the actuators and the robot body, a goal attainable by implementing transducers that exploit the optical, rather than the electrical, conductivity of sensing materials. In fact, the monolithic integration of optical sensing set just one requirement, i.e., the material of both sensors and robot must be transparent to light. This feature, even if challenging, is still less constraining than those required from other transduction mechanisms, which may imply the addition of functional fillers (e.g., magnetic, electrically‐conductive) stiffening the robot and creating bi‐material interfaces. As a first step, our previous work introduced the Monolithic Perceptive Unit (MPU): a fully 3D‐printed lattice cell in which the constituent elastomer itself functions as an optical sensor [23].

Lattice architectures have emerged as a promising alternative to bulk soft bodies. Although bulk material functionalization is possible, it often comes at the expense of mechanical performance [24]. In contrast, lattice architectures retain the softness of the bulk material while offering internal pathways and anchor points, which simplify the integration of actuators and sensors and enable support‐free fabrication by creating in situ supports during 3D printing. For example, tendon‐driven lattices have been used to reproduce musculoskeletal behaviors by routing cables through the structure [25, 26]. Alternatively, we have previously introduced lattice‐embedded actuators which can achieve bending and jointless behavior [27]. In this work, an IWP‐TPMS (triply periodic minimal surface) lattice is adopted. While the underlying topology defines the deformation modes, the stiffness ‐in addition to the intrinsic material properties‐ depends on the cell dimensions. Since the lattice serves as a compliant medium for the embedded actuators, and it must enable the gripper to extend, bend, and conform around objects during grasping, a low bending stiffness of the chosen lattice configuration is pursued.

The draw of the proposed monolithic method in soft robotics lies in simplifying the design process: using a single‐material body that combines actuation and sensing without post‐processing. However, few materials are capable of delivering all required functionalities (i.e., flexibility, printability, sensing) while remaining compatible with streamlined fabrication. Recent advances in commercial 3D printing [28] have enabled one‐step processes to create hybrid systems, yet fully printed monolithic soft systems are still rare. Existing “monolithic” soft grippers generally do not integrate sensing into the same continuous material domain: some include no sensing at all [29], others rely on multimaterial capacitive layers [30], rigid pressure sensors embedded post‐printing [31], or pressure‐based feedback from pneumatic channels [32]. These approaches either introduce material mismatches or limit feedback to internal air‐channel deformation, and none employ lattice architectures for co‐fabricated sensing and actuation. In contrast, MELEGROS integrates optical sensors, actuators, and structure in a single‐material lattice body, eliminating interfaces and supporting simulation‐guided placement of embedded sensors.

**FIGURE 1:**
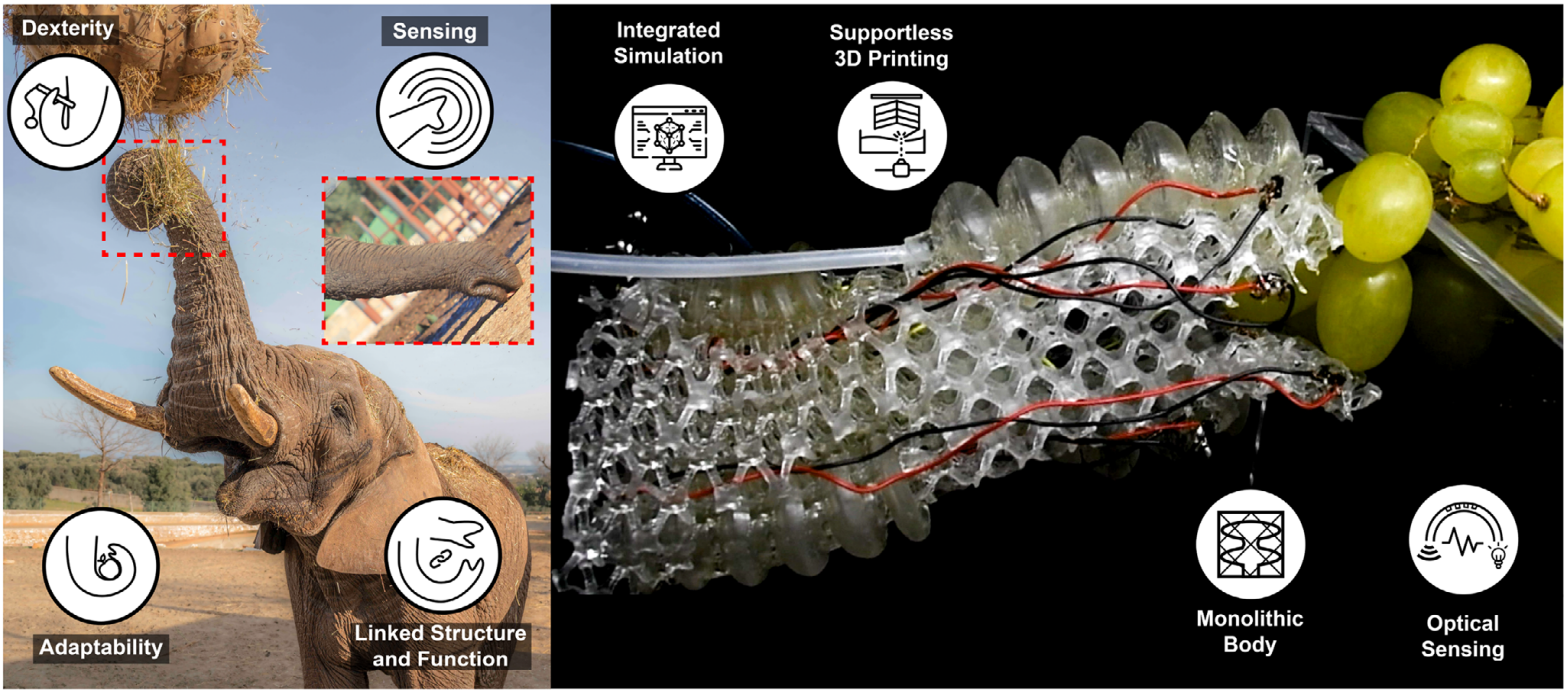
The MELEGROS concept inspired from the elephant trunk. Left, photograph (courtesy of Matteo Montenero) of a male African elephant (ZooSafari, Italy), where the distal region of the trunk is highlighted. Right, the continuous structure of the artificial elongating gripper monolithically embedding optical sensing, fluidic actuation and lattice‐based body.

Inspired by the morphology and behavior of the distal region of the African elephant trunk [2, 4], and enabled by an architected design, we introduce the MELEGROS concept: a **M**onolithic **Ele**phant‐Inspired **Gr**ipper with **O**ptical **S**ensors (Figure 1). The system is built from a soft lattice with smoothly connected bladder‐shaped actuators, which not only allow the system to elongate, compress, and bend, but act as the body of both gripper and soft optical sensors. The design does not aim to mimic natural muscular arrangements, but rather focuses on functional integration of actuation and sensing. To achieve this objective, and building on our recent results in simulating soft lattice structures [33], our method is based on a workflow linking design and fabrication through simulation in SOFA (Simulation Open Framework Architecture) [34, 35] to design and position the soft optical sensors. The output is a fully‐integrated design that is fabricated via a single 3D‐printing process. In this work, we focus on investigating the sensing functionality by targeting the discrimination of exteroceptive from proprioceptive information during grasping tasks. We show how the specific monolithic architecture enables MELEGROS to perform enveloping grasps and delicate pinching maneuvers (extending its functionality beyond simple parallel‐jaw closure) and to reach and bend independently in an intrinsic workspace, where a specific object can be reached from multiple directions before being grasped, similar to the natural model.

## 2 Results

### 2.1 Design of the Gripper

The design process begins with two key steps that establish the basis for monolithic integration. First, the lattice geometry is selected and characterized through mechanical testing (Figure S2), providing homogenized stiffness values for use in simulation. Second, a half‐embedded actuator is fabricated to validate printability and assess kinematics during actuation (Figure S6). These preliminary results informed the integration of actuators within the lattice and guided the simulation of the complete gripper presented in the following sections.

To address monolithic integration, we implement an iterative workflow that links design (of structure, sensing, actuation) and fabrication through simulation (Figure S1). At the core of the monolithic approach is the lattice which has three main roles. First, it enables monolithic fabrication of sensors and actuators. Its struts and voids provide a scaffold for the waveguides and the actuators, which can be printed all together without supports. This leads to a monolithic architecture and reduces post‐processing. Second, it enables full compliance and adaptability during grasping. Owing to its TPMS geometry, the lattice can deform laterally, allowing the gripper to conform around objects of varying shapes (e.g., cube, sphere, star) and sizes (in the range of 12.5 to 25 mm) while maintaining contact along multiple surfaces. This flexibility supports both enveloping grasps and delicate pinching, enabling interaction with a wider variety of objects than simple parallel‐jaw closure would allow. Third, it transmits the deformation of the embedded longitudinal actuators, without critically limiting the elongation of the whole gripper and enabling reaching.

An IWP‐type TPMS geometry (Equation S1) is adopted for the lattice, which reduces bending stiffness (w.r.t. a same‐size bulk solid) while maintaining structural continuity, allowing the distal arm and the tip to deform as a single continuous unit, mimicking the bioinspired integration observed in elephant trunks. The lattice is designed to be compliant and to enable supportless 3D printing, while accommodating embedded optical sensing and serving as the body of the gripper. Strut thickness is chosen (1.5 mm) to maintain flexibility while supporting the integrated waveguides. The selected cell size is determined through iterative prototyping to achieve printability with sufficient compliance (Young's modulus < 1 MPa) [36, 37]. The chosen cell size (12.5 mm) determines the minimum object size that can be grasped without slipping within the lattice air cell (∼8 mm void) while attached to the actuators (Figure S4). To evaluate its mechanical response, cubic lattice samples are subjected to compression stress–strain tests, demonstrating nonlinear stiffness and recoverable deformation (Figure S2). These results are used to inform the simulations (described in later sections), where the response is linearized up to 40% strain and approximated by an effective Young's modulus of ∼12 kPa. This modulus is applied to the design's bulk volume envelope (Figure S19) as the homogenized material property for the lattice.

Actuators capable of elongation and contraction are selected, so when they are printed half‐embedded by the lattice, the anchor points introduce strain‐limiting behavior, which enables the actuators to be repurposed for bending (Figure S6). Bladder‐like chambers are chosen because their circular cross‐section allows direct attachment to the TPMS nodes, while their convoluted geometry accommodates local expansion at lattice anchor points and compression of the intervening voids. The pneumatic actuators are implemented as bladder‐like chambers arranged in series and co‐designed with the lattice anchor points to ensure symmetric deformation between chambers.

**FIGURE 2:**
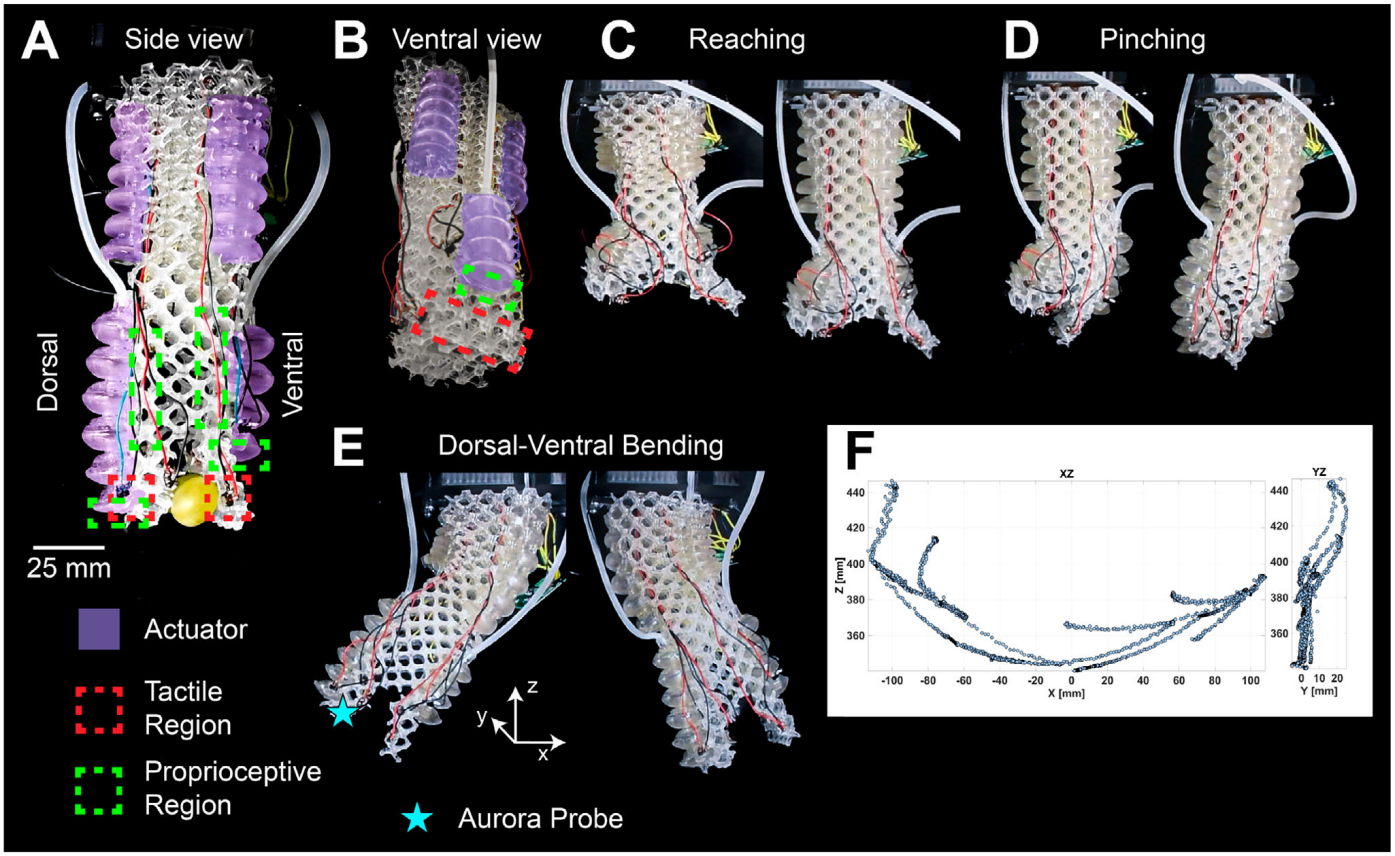
Overview of MELEGROS design and motions. (A) Side and (B) ventral views of MELEGROS depicting bladder‐like actuators in purple (dorsal and ventral fingers have an actuator with 6 and 4 chambers, respectively; proximal region integrates three 6‐chamber actuators), with sensor regions of interest indicated by red and green rectangles. MELEGROS during (C) reaching, (D) pinching, and (E) dorsal‐ventral bending. A star indicates where the electromagnetic probe (AURORA, Northern Digital Inc., Canada) was placed for defining the (F) workspace boundaries defined as *x* ∈ [− 113, 107] mm, *y* ∈ [− 5, 25] mm, *z* ∈ [340, 446] mm, captured sweeping across dorsal and ventral bending.

The gripper design is conceptually inspired by the elephant trunk tip, whose asymmetric finger‐like projections achieve versatile grasping behaviors (Figure S7). Similarly, our system integrates two opposing lattice‐supported actuators at the distal end of the structure, with the dorsal and ventral fingers having 6 and 4 chambers, respectively (Figure 2). Each actuator is embedded along the lattice struts in such a way that pressurization induces bending toward the central axis of the gripper. Three 6‐chamber actuators are placed proximal (i.e., at the base) of our design to achieve contraction, elongation, and bending. These proximal actuators are arranged with one on the dorsal side along the central axis, and two on the ventral side resembling the natural “ventral ridge” [38, 39]. The objective herein is not to mimic the natural muscular arrangements [2], rather the placement of the actuators is determined to achieve some bioinspired behavior like the reaching, pinching, and dorsal‐ventral bending, enabling independent reach and bending motion modalities. This way MELEGROS can move in a space suitable for approaching objects from multiple directions without their precise pre‐alignment with the tip, as will be shown later.

Functionally, the asymmetric actuator arrangement is intended to mimic some basic motions of the distal trunk (Figure 2). When the proximal actuators are driven together, the surrounding lattice compresses and expands to achieve reaching (Figure 2C). These proximal actuators are also capable of moving independently, resulting in dorsal and ventral bending (Figure 2E). The finger actuators can be driven asymmetrically, one finger bending inward while the other remains passive, enabling pinch‐like grasping (Figure 2D) of multiple objects (Figure S21). Additionally, MELEGROS (m = 132 g) can drive its fingers together to grasp and lift an object weighing twice its own mass (cylinder: L = 120 mm, d = 30 mm, m = 264 g) (Video S1).

### 2.2 Sensing Design and Simulation

**FIGURE 3:**
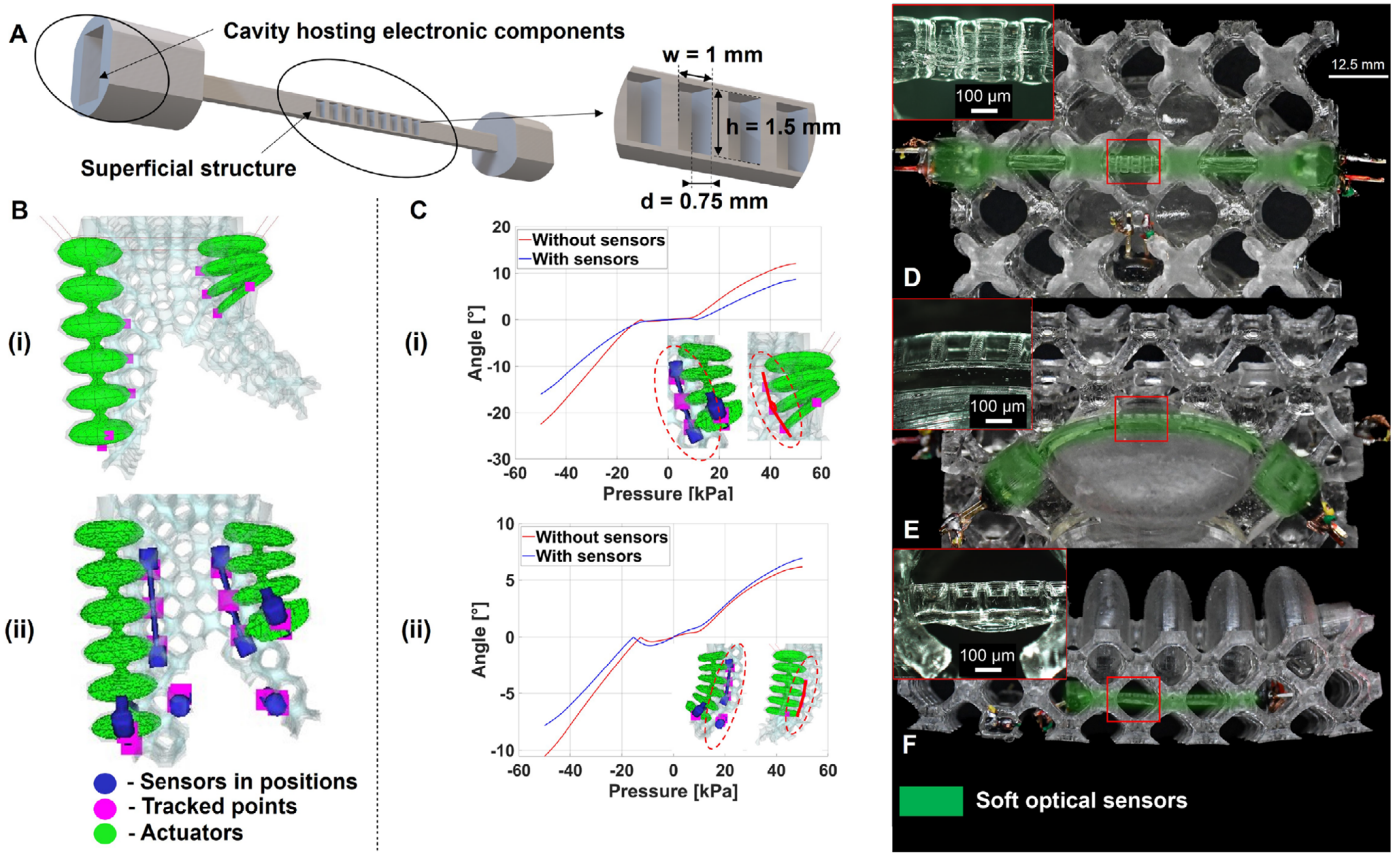
Design, simulation, and placement of optical sensors. (A) Design of the optical sensors and their superficial structures (width = 1 mm, height = 1.5 mm, depth = 0.75 mm). (B) Simulations with tracked points within (i) Model A: sensor regions of interest, and (ii) Model B: incorporated sensor structures. (C) Simulated finger bi‐directional bending angles from ‐50 to 50 kPa to inform design and placement for (i) ventral bending and (ii) dorsal bending sensors. (D) Tactile sensor (37.5 mm long), (E) pressurized actuator sensor (25 mm long), and (F) finger bending sensor (37.5 mm long) highlighted in digital microscope images.

Sensing in MELEGROS is focused on measuring bending of the fingers, deformation in fluidic chambers, and tactile response. Optical waveguide sensors are used (which we introduced in a previous work [23]): 3D‐printed polymer waveguides incorporating a surface pattern (i.e., a series of wells) that amplify bending sensitivity (Figure 3A). In brief, an LED emits light into the waveguide toward a photodetector. Bending increases optical losses, primarily via scattering from the patterned surface, thereby reducing the received intensity and producing a corresponding decrease in the photodetector voltage. The general waveguide design for both proprioceptive and tactile sensors is provided in Figure S9, aimed at producing a sensing response due to actuator motion and external contact, respectively.

In comparison to previous work, a new parametrization of the sensor design is implemented to enable monolithic fabrication. Specifically, adjustments are made to the thickness of the waveguide and the geometry of the surface pattern (Figure S8A). Sensor thickness is constrained by two factors: waveguide printability and the printer's nominal resolution (50 µm). Additionally, the waveguide must be sufficiently thick to accommodate wells of adequate depth without compromising durability under repeated loading. Given that the minimum well depth to obtain a reliable print is 0.5 mm, the waveguide thickness is set to 1.5 mm, which also establishes the aforementioned lattice strut thickness. The dimensions of the surface pattern are defined by maximizing linearity and sensitivity, by using COMSOL Multiphysics simulations (Figure S8A). Parametric sweeps of the pattern geometry yield a well depth d = 0.65 mm and width w = 1.0 mm. Further details are provided in Figure S8B. Then, sensor placements are established to ensure output signal fidelity while preserving mechanical compliance. This is guided by simulated position trajectories, targeting regions that consistently undergo contact with objects during typical finger motions (for tactile sensing) and regions that experience bending trajectories independent of contact (for proprioception).

A homogenized material model was assigned to the simulated gripper based on compression tests of lattice samples, and all components were meshed and actuated using a scripted workflow. Pneumatic membranes and optical waveguides were treated as distinct elastic subdomains, and time‐varying pressures were applied to the cavities to evaluate their deformation and sensing trajectories. A full description of the material parameters, meshing procedures, and pressure‐control algorithms is provided in the Supporting Information.

The methodology begins with defining an initial gripper design (informed by the lattice printability trials) in simulation. Observations of the half‐embedded actuator movements provide early feedback on the potential design and placement of the optical waveguides in the simulated gripper. Then, the sensing regions of interest are defined (Model A: Figure S13). The following steps consist of defining some points in these regions based on waveguide geometry (Figure 3B(i)) and measuring their position trajectory during actuation (from ‐50 to 50 kPa). If the displacement is sufficient to warrant a sensing response, then waveguides are added to the robot model within the simulation environment (Model B: Figure S14 and Figure 3B(ii)). Consequently, the simulation framework Model B is used until some specific conditions are fulfilled. First of all, the variation of angle in the actuator‐waveguide system must confirm that it is possible to get a sensing response in the real prototype. Additional consideration for the sensors' placements must be given to the printability (using the lattice nodes as support elements of the waveguides). Indeed, if the waveguide behavior is decoupled (e.g., tactile waveguide bending < 5° during finger bending shows a small influence of general movements on tactile sensors) and if the integrated waveguides do not significantly change the mechanical behavior of the gripper (e.g., change in bending angle < 5° doesn't affect the task performance), the iterative process is finished. This analysis, critical for distinguishing sensing signals deriving from touch from those induced by gripper motion, is made possible by the monolithic architecture, which removes material interfaces between sensors and actuators and thereby reduces model uncertainty.

According to these requirements, the tactile and proprioceptive regions of interest are defined as depicted in Figure 2A, and the finalized sensor layout is shown in Figure 3D–F. Specifically, a total of six soft optical waveguides are placed as follows: (i) a tactile sensor is on the inner surface of each finger (Figure 3D); (ii) a sensor is on the last chamber of each finger actuator, to sense the fluidic deformation (Figure 3E); and, (iii) a sensor is along the length of each finger, to sense the bending motion (Figure 3F). Because the dorsal and ventral fingers contain actuators with six and four chambers, respectively, the sensor lengths follow this asymmetry, with the ventral finger incorporating a shorter bending sensor.

Herein, we explain how this layout allows for satisfying all the aforementioned requirements. The bending angles of the sensor trajectories observed in the simulation (Figure 3C) reach up to 40°, which we previously demonstrated to be sufficient for generating a sensing response for this type of sensor [23]. This is further validated in Section 2.3, where the sensor response is characterized in the half‐embedded actuator integrating the three sensor types presented in this work. To minimize cross‐talk, bending sensors are placed along segments of observed curvature (close to the actuators, as shown in Figure 2) that remain contact‐free from the objects due to their position inside the lattice. Tactile sensors are instead positioned on the inner finger surfaces where contact is expected during grasping.

The results reported in Figure S20 confirm that the simulated bending of tactile waveguides is negligible during the opening and closing phases of the gripper, providing strong hints on the possibility to decouple proprioceptive and tactile feedback in the real prototype. Finally, the interaction between the compliant lattice and the regions stiffened by sensor inclusion was represented to verify the effect of the waveguides on the mechanics of the gripper. The presence of sensors (waveguides) locally increases the rigidity of the structure and thereby alters its deflection response (Figure 3B). Figure 3C illustrates this effect: when sensors are not included, the actuator exhibits larger angular deflections under the same pressure, whereas adding the sensor domains leads to slightly reduced angles. This outcome confirms the expected result that sensors contribute mechanical stiffness to the system, an effect that must be taken into account to correctly predict their position trajectories.

To further examine the sensing regions, 2D projections of sensor‐point trajectories are analyzed (as described in the Supplementary Information). For example, for Model A, the ventral finger modes **open2** and **close2** (Figures S15 and S16) reveal that, even in the absence of embedded sensors, the monitored points trace smooth and consistent paths over the full actuation cycle, confirming their suitability as proprioceptive sites. For Model B, the corresponding trajectories during the simultaneous‐finger modes **open** and **close** (Figures S17 and S18) further demonstrate that integrating the sensors has a negligible impact on overall motion while preserving distinct trajectories for angular readout.

Eventually, the final monolithic prototype is successfully printed. The integration between waveguides, lattice, and actuators is shown in Figure 3D–F. The optical waveguides (highlighted in green) are co‐printed with the gripper, using the lattice and the actuator chambers as in situ supports. Magnified insets display the surface patterning across sensor types, confirming high fidelity in small‐feature fabrication.

### 2.3 Characterization and Sensing Response

**FIGURE 4:**
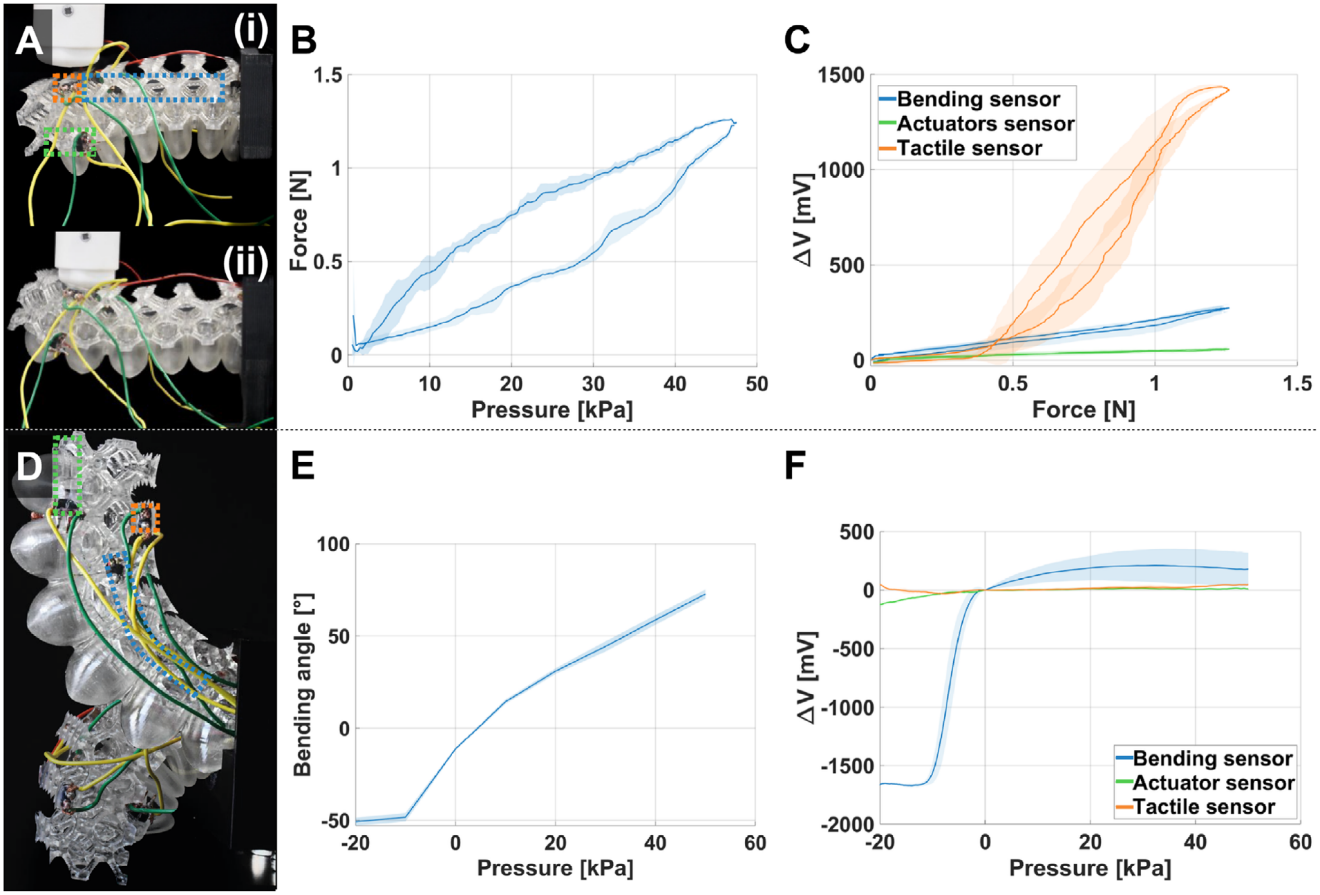
Characterization of sensorized half‐embedded actuators. (A) Blocking force tests of the half‐embedded bladder‐like actuators at (i) rest, and (ii) actuated state. (B) Resultant force data from the triaxial load cell, and (C) corresponding sensor responses. (D) Free bi‐directional bending of the half‐embedded actuator with (E) recorded angles were captured from ‐20 to 50 kPa in ImageJ, and (F) corresponding sensor responses. Sensor positions are outlined in panels A and D in their respective colors, and detailed images of the final version of the sensors are shown in Figure 3D–F. Shaded areas of graphs represent ±1 standard deviation (N = 3).

Following the simulation informing the final optical sensor placement, two prints can be fabricated. The first is the sensorized version of the half‐embedded actuator, used for the actuator's characterization and preliminary check of sensors' response, since its movement resembles that of MELEGROS. The second is MELEGROS, which is used for grasping applications to assess sensor fidelity. For the sensorized half‐embedded actuators, experimental tests are conducted within a pressure range of –20 to 50 kPa, as lower pressures (below –20 kPa) did not produce additional bending. Within this range, bending angles and blocking forces are observed to scale with applied pressure (Figure 4). Half‐embedded actuators achieve free bending angles from –50° (–20 kPa) to 75° (50 kPa). Under positive pressure, half‐embedded actuators expand to produce a blocking force of 1.25 N (50 kPa).

Sensor responses are collected on the same half‐embedded actuators under these blocking‐force and free‐bending tests (Figure 4). Under blocking‐force conditions (Figure 4A–C), the tactile sensor (orange) produced the largest response (ΔV ≈ 1.4 V), the bending sensor (blue) an intermediate response (ΔV ≈ 0.25 V), and the actuator sensor (green) the smallest (ΔV ≈ 0.06 V); each dataset comprises three cycles. During free bending experiments (Figure 4D–F), the bending sensor exhibited the largest response (ΔV ≈ 2.0 V), and its voltage trajectory resembles the measured bending angle. The distinct response profiles indicate effective decoupling between tactile and proprioceptive information.

Following the characterization of the previous lattice/actuator/sensors structure, MELEGROS is evaluated through grasping trials to assess sensor responses during these tasks. The asymmetric structure of the tip enables a bioinspired grasping technique, in which the ventral finger can remain passive, while the dorsal scoops objects. Due to this capability, MELEGROS can successfully grasp and lift not only the aforementioned heavy (double its weight, 264 g) and elongated objects (cylinder: L = 120 mm, d = 30 mm), but also small objects of various shapes, such as spheres, cubes, and stars with different dimensions (d = 12.5, 25 mm) (Figure S21). Moreover, grasping of multiple objects is shown and achieved owing to the adaptability of the lattice structure.

**FIGURE 5:**
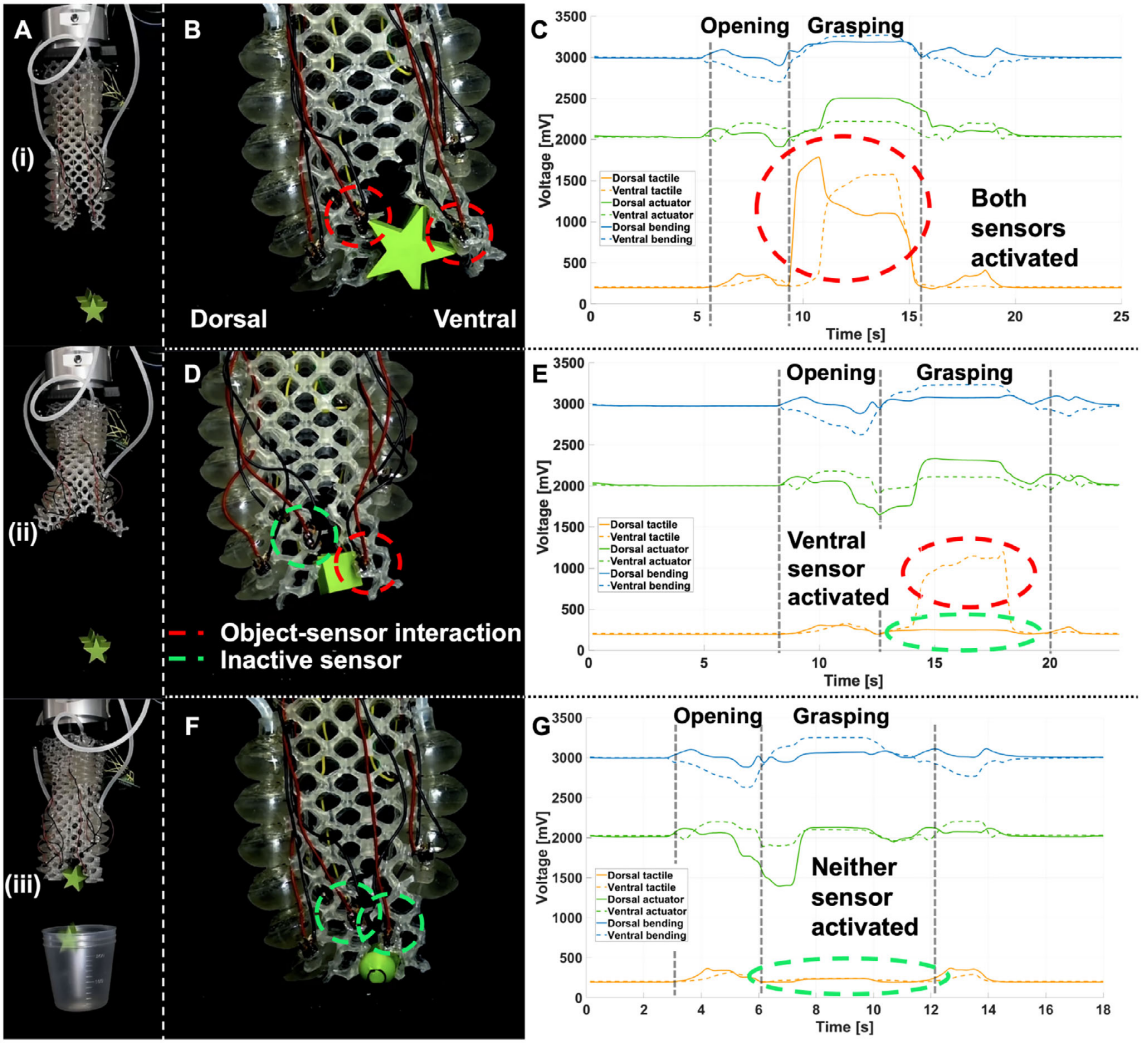
Object Grasping and Sensor Response in MELEGROS. (A) MELEGROS (i) approaching, (ii) grasping, and (iii) releasing multiple objects (three stars, 12.5 mm). Grasping scenarios where sensors are activated in (B–C) both fingers (two stars, 25 mm), (D–E) only the ventral finger (three cubes, 12.5 mm), and (F–G) neither finger (three spheres, 12.5 mm).

Furthermore, MELEGROS is installed on a robotic arm (UR5e, Universal Robots, Denmark) to perform pick‐and‐place tasks with different objects (Video S2) while recording the response of the sensors. The sensing response is influenced by the different movements and sizes of the objects (Figure 5). The behavior of the sensorized gripper can be summarized as follows: from a relaxed state, it contracts along its axis and opens the fingers (Figure 5A(i, ii)), the robot arm approaches the target, MELEGROS elongates, and the fingers close. These phases elicit distinct signals: actuator and bending sensors report chamber pressurization and finger deformation during opening/closing, whereas tactile sensors respond primarily upon object contact. Following this, the robot arm moves above a cup, MELEGROS opens, and the object is released (Figure 5A(iii)). Across experiments, all the sensors exhibit reproducible patterns. Owing to gripper asymmetry, tactile responses vary with respect to object size (Figure 5B–G): larger items (e.g., 25 mm star shapes) activate both tactile sensors on the dorsal and ventral fingers (Figure 5B,C), whereas smaller items activate only one sensor or none (Figure 5D–G). During the grasping of three small cubes (Figure 5D,E), only the ventral finger comes in contact with the objects and is activated. For the case of the small spheres (Figure 5F,G), neither tactile sensor is activated, as their capture does not laterally deform the lattice.

### 2.4 MELEGROS in Action

**FIGURE 6:**
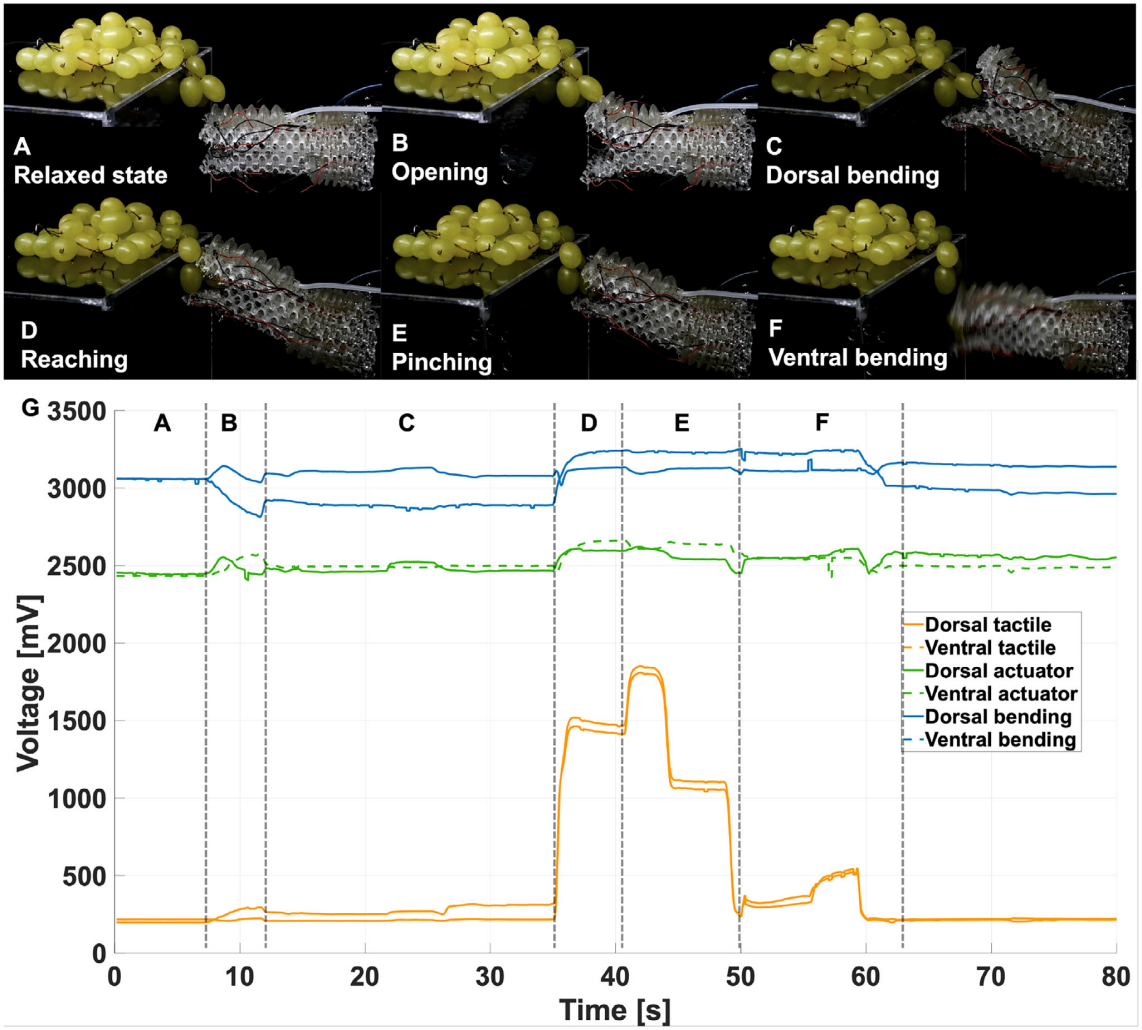
MELEGROS is approaching a bunch of grapes from the side, with corresponding sensor data for all six sensors. MELEGROS in (A) relaxed state, (B) opening, (C) dorsal bending, (D) reaching toward and coming into contact with the grape, (E) pinching the grape, and (F) ventral bending (Video S3).

Conventional soft grippers primarily execute pinch grasps and function solely as end‐effectors. In contrast, MELEGROS can bend and extend independently, providing improved freedom of motion to approach an object from multiple directions and to grasp it without requiring a precise pre‐positioning of the same. Crucially, the gripper performs these manoeuvres while delivering motion‐specific sensory readouts. Figure 6 and Video S3 depict dexterous reaching and grasping of delicate fruit (grapes). In this scenario, the gripper is positioned near the target and opens from a contracted state (Figure 6A,B), performs dorsal bending (Figure 6C), reaches by elongating ventral actuators to contact the grape (Figure 6D), pinches the grape (Figure 6E), and then bends ventrally to detach/pick the grape from the stem before repositioning and opening to release (Figure 6F).

Moreover, the sensors respond to each phase of the motion, as shown in Figure 6G. In the relaxed state (A), the sensory signals remain constant. During opening (B), bending and actuator sensors in each finger are activated. When the base bends dorsally without finger movement (C), sensor outputs remain unchanged. In the reaching and closing phase (D), both bending and actuator sensors are activated again, but with a distinct response compared to the “simple” closing (empty state), while the tactile sensors confirm contact with the object. During pinching and picking (E), bending and actuator sensors remain constant, but tactile sensors register a sharp peak as force is applied to detach the grape, followed by an immediate drop once it is removed. Finally, during ventral bending and opening (F), bending and actuator sensors provide steady feedback during base bending, then return to the values associated with opening. Tactile responses decrease after picking, reflecting the reduced contact force, and ultimately return to relaxed‐state values.

## 3 Discussion and Conclusion

This work advances soft robotics design by demonstrating a monolithic strategy in which actuation and sensing are co‐designed, co‐fabricated, and co‐located within a single lattice body. Unlike conventional and existing monolithic approaches [40] that depend on trial‐and‐error prototyping, we establish a simulation‐reinforced workflow in which tracked position trajectories predict how sensors and actuators interact within the body. Noteworthy, we use simulation as a design tool (rather than a post hoc validation step) to address a long‐standing challenge in soft robotics: the decoupling of tactile and proprioceptive feedback within a single compliant body.

The main aspects of our work deserve emphasis as generalizable contributions. The presented monolithic approach joins sensing and structure, and proves the possibility of building continuum manipulators where the arm and the end‐effector form a continuous structure. Mimicking such basic feature of the natural distal trunk gives rise to a new concept of a soft robotic manipulator.

Indeed, the proposed lattice architecture enables support‐free printing of complex compliant features and internal cavities, eliminating sacrificial materials and post‐processing. Then, integrating actuators within the compliant lattice body, MELEGROS is able to reach, bend, grasp, and release objects (singular or multiple) within a workspace. The results of the preliminary grasping and manipulation experiments are promising and suggest that our work goes beyond other approaches, resembling the elephant tip, which rely on rigid robotic arms to navigate the workspace [41, 42, 43]. Moreover, crucially, tactile and proprioceptive information are provided by the body itself, serving as the transduction medium, removing internal interfaces and collapsing the distinction between “robot” and “sensor”, reinforcing our central premise. Our findings demonstrate that MELEGROS can be considered for a broad range of real‐world applications in typical environmental conditions.

Finally, but not less important, reliable simulation is enabled by the material continuity and gives important insight on sensing region of interest trajectories to tune sensors' geometry and placement before printing, turning what is usually iterative trial‐and‐error into a targeted, more directed design step. Therefore, only a few different prints in total are required in this entire process: the half‐embedded actuator and lattice before sensor placement, and the actuator for characterization and gripper after sensor integration.

Building on these results, the next phase will advance MELEGROS toward robust closed‐loop manipulation. The embedded sensors enable feedback control, a defining capability for robotic systems. Priorities will include quantitative investigation of payload limits, implementation of closed‐loop control with phase‐resolved feedback, and long‐duration testing to characterize durability and drift. In parallel, graded materials [26] and interpenetrating lattices [25, 44] within the robot's body will be explored to extend functionality and deepen bioinspired design. In particular, these strategies open the possibility of introducing stiffness gradients within a monolithic body. Such graded architectures would support multiple movement and sensing modalities, moving beyond current manipulators while echoing the functional and morphological continuity of the natural trunk. As the field moves toward hybrid soft‐rigid grippers for enhancing payload capacity [45, 46], it is noteworthy that MELEGROS achieves a payload of twice its system weight while remaining a fully monolithic soft structure. This highlights the need for further systematic evaluation of payload capabilities in soft monolithic systems across different application contexts. Finally, scaling via larger build volumes or modular printing will support a full trunk with further elephant‐inspired modalities such as wrapping [2] and twisting.

## 4 Experimental Section

### 4.1 Material and Printing Parameters

All soft structural components were fabricated using Elastic 50A Resin (Formlabs, United States), a flexible photopolymer suitable for high‐strain applications. Samples were printed using a Form 4 (Formlabs, United States) stereolithography (SLA) printer with a layer thickness of 0.1 mm. Exposure parameters were as follows: perimeter, model, and support fill regions received an energy dose of 38.40 mJ/cm^2^, whereas the light intensity was set as 11.5 mW/cm^2^.

Post‐processing was carried out using the standard Formlabs protocol. Printed parts were washed in isopropyl alcohol for 20 min using a Form Wash station, followed by UV curing at 70°C for 30 min in a Form Cure unit to ensure complete polymerization.

### 4.2 Characterization Specimens and Protocol

To evaluate the mechanical response of the integrated soft structure, both passive lattice and active actuator configurations were tested under mechanical and pneumatic loading conditions. Compression and bending tests were performed to assess deformation behavior, hysteresis, and actuation capabilities under cyclic conditions. Blocking force measurements were conducted to quantify the actuator's output under constrained conditions.

Cubic lattice samples measuring 62.5 mm per side were fabricated using an IWP‐type TPMS geometry with 12.5 mm unit cell size and 1.5 mm wall thickness. Each sample (N = 5) was tested using a Universal Testing Machine (Zwick/Roell, Germany) at a constant rate of 10 mm/min following a 1 N preload. Compression was applied to 20%, 40%, and 60% strain to evaluate nonlinear stiffness and hysteresis.

Bladder‐like actuators composed of six serially connected pneumatic cells (Figures S3 and S5) were embedded halfway into matching lattice volumes (Figure S6). Actuators (N = 3) were driven by sinusoidal pressure inputs ranging from ‐20 to 50 kPa. Bending deformation was recorded on video and analyzed using an image processing program (ImageJ [47]). For blocking force measurements, the actuators were mounted on a micrometric translation stage (M‐111.1DG, Physik Instrumente, Germany) interfaced with a triaxial load cell (ATI Nano 17, ATI Industrial Automation, United States). A half‐wave rectified sine pressure input of up to 50 kPa was used to assess peak force generation at maximum extension. For free bending measurements, the actuators were mounted to an optical table (Standa, Lithuania) while actuators were driven with pressures from ‐20 to 50 kPa in increments of 10. Sensor data was recorded over 100 cycles during both blocking force and free bending tests to assess repeatability (Figure S10), as well as for 20 min during opening and closing of the gripper (Figure S11). Images were taken with a digital camera (Nikon D7500, Nikon, Japan) and bending angles were analyzed in ImageJ.

To map the MELEGROS workspace boundaries, an electromagnetic probe (AURORA, NDigital, Canada) was placed at the tip of the dorsal finger (Figure 2E) as movement swept all modes of reaching, pinching, and dorsal‐ventral bending.

### 4.3 Simulation of Integrated Lattice

The simulation of MELEGROS was supported by a meshing workflow that converted CAD geometries into finite element models for SOFA simulations. STEP files defined the lattice volume, membranes, and cavities. In the sensorized configuration, additional STEP files specified the optical channels. All parts were meshed in Gmsh [48] through a Python interface. The workflow involved three stages: importing the geometry and removing duplicates, fragmenting overlapping volumes, and generating a tetrahedral mesh. The final meshes were exported in VTK format.

A subsequent procedure merged the separate component meshes into a single unstructured grid. This was achieved by detecting nodes within a specified tolerance and consolidating them, thereby ensuring a consistent connectivity across membranes, cavities, and lattice volume. The resulting merged mesh provided the mechanical basis for the simulator, where material properties were assigned according to homogenization tests.

Within the simulator, the homogenized properties of the lattice, identified from representative element compression tests, were assigned to the global volume. The pneumatic membranes were treated as deformable regions mapped to the volume, while the internal cavities defined pressure application surfaces. This arrangement enabled five independent pneumatic subsystems: three at the base, producing reaching and bending, and two at the tip, responsible for opening and closing. In the sensorized variant, the optical waveguides were introduced as elastic subdomains with higher stiffness than the surrounding lattice, allowing the positions of interest to be tracked during movement.

Control logic was implemented through dedicated scripts that read time‐varying pressure inputs, applied them to the cavities, and monitored structural responses. Monitors tracked nodal displacements, contact interactions with rigid objects, and reaction forces in predefined regions of interest. The framework also allowed interactive actuation through manual commands, enabling exploration of different loading scenarios in real time. This integrated modeling framework allowed testing of actuation strategies and evaluation of the mechanical influence of the embedded sensors.

The detailed implementations and simulation results are provided in the Supporting Information. Specifically, Algorithm one outlines the scene construction pipeline, while Algorithm two defines the pressure control logic. Two complementary models were realized: the *Sensor‐Position Model* (Model A; Figure S13), which specifies candidate sensor sites, and the *Sensor‐Integrated Model* (Model B; Figure S14), in which sensor bodies are mechanically embedded. To visualize sensor trajectories, Figures S15 and S16 report 2D projections for Model A under the ventral‐finger modes **open2** and **close2**, whereas Figures S17 and S18 illustrate the corresponding kinematics for Model B under simultaneous‐finger modes **open** and **close**. These graphs form the basis for Figure 3, linking sensor trajectories to the extracted angular variations. In addition, Figure S19 provides a broken‐out view of the manipulator, highlighting the lattice, membranes, pneumatic cavities, sensors, and envelope considered in the simulations, while Figure S20 presents the angle–pressure relationships of two integrated tactile sensors, emphasizing the influence of sensor positioning on the measured angular response.

### 4.4 Sensing Setup

The photoemitters used in the devices (infrared LEDs, VSMY1850) have a peak emission at 850 nm, corresponding to the peak reception of the photoreceivers (TEMT7100X01). Signals were acquired using a custom‐designed PCB (Figure S12). The PCB enables the sequential activation of the emitters, which minimizes interference from ambient light. The emitters are modulated at 200 Hz, allowing the system to record the ambient light level during the “off” phase and subtract it from the measurements taken during the “on” phase. In addition to the transistor circuitry, the PCB incorporates tunable resistors (TC33X‐2‐202E), which allow adjustment of receiver sensitivity and emitter power to accommodate variations in sensor geometry and mechanical deformation. The tunable resistors enable calibration of the system so that all sensors exhibit a uniform baseline response. This compensates for small variations introduced during the manufacturing process, ensuring that such discrepancies do not significantly affect the overall sensor performance. Data collection was done with Python 3.13, and data analysis was performed using MATLAB (The MathWorks, Inc.). Cavities at the two ends of each waveguide were designed to match the size of the components. LEDs and transistors were dipped in the resin Elastic 50A, then placed inside the cavities. Then, all components were UV‐cured for 2 min. Once the components were fixed in position, an additional drop of resin was dropped and UV‐cured to seal the cavities and ensure robust embedment.

### 4.5 Design of Pneumatic Actuators and Control Setup

To control the pneumatic actuators of MELEGROS, an I/O Device (NI USB‐6218, National Instruments, United States) was used, connected to a simple LabVIEW (National Instruments, United States) program. A dedicated analog output was attached to positive (ITV‐0010, SMC, Japan) and negative (ITV‐0090, SMC, Japan) pressure regulators to control the actuation pressure. The outputs of the regulators were attached to a 3/2 solenoid (V114A‐5LOU, SMC, Japan) for each bladder. Each bladder was interfaced with a 2‐port solenoid valve (VDW20GA, SMC, Japan) to maintain pressures while switching bladders. A dedicated pressure and vacuum (VCP 80/VCP 130, VWR, United States) was used during operation.

### 4.6 Statistical Analysis

All sensing data were pre‐processed by subtracting the initial baseline voltage to obtain Δ*V* for each sensor. For cyclic loading‐unloading tests of the sensorized half‐embedded actuators, three actuation cycles were recorded per condition, and Δ*V* was computed for each cycle. Data are reported as mean ± standard deviation (SD) over these cycles. For the cube compression tests used to determine the homogenized lattice properties, five lattice samples (*n* = 5) were tested; stress–strain curves are presented as mean ± SD. No formal hypothesis testing or between‐group statistical comparisons were performed; all statistics are descriptive and intended to demonstrate repeatability and variability across samples. All data processing and statistical computations were performed in MATLAB (MathWorks).

## Author Contributions

Petr Trunin, Diana Cafiso, and Anderson Brazil Nardin contributed equally to this work. **Petr Trunin**: Writing – review and editing, Writing – original draft, Visualization, Validation, Methodology, Investigation, Formal analysis, Data curation, Conceptualization. **Diana Cafiso**: Writing – review and editing, Writing – original draft, Visualization, Validation, Methodology, Investigation, Formal analysis, Data curation, Conceptualization. **Anderson Brazil Nardin**: Writing – review and editing, Writing – original draft, Visualization, Validation, Methodology, Investigation, Formal analysis, Data curation, Conceptualization. **Trevor Exley**: Writing – review and editing, Writing – original draft, Visualization, Validation, Methodology, Investigation, Formal analysis, Data curation, Conceptualization. **Lucia Beccai**: Writing – review and editing, Supervision, Resources, Project administration, Methodology, Funding acquisition, Conceptualization.

## Conflicts of Interest

The authors declare no conflicts of interest.

## Supporting information


**Supporting File 1**: advs73518‐sup‐0001‐SuppMat.pdf.

**Supporting File 2**: advs73518‐sup‐0002‐Video_S1.mp4.

**Supporting File 3**: advs73518‐sup‐0003‐Video_S2.mp4.

**Supporting File 4**: advs73518‐sup‐0004‐Video_S3rev.mp4.

## Data Availability

The data that support the findings of this study are available from the corresponding author upon reasonable request.
