# Supplementary material for: MELEGROS: Monolithic Elephant‐Inspired Gripper with Optical Sensors
Source: Adv Sci (Weinh). 2026 Feb 2;13(22):e18878. doi: 10.1002/advs.202518878 (PMC13088296; doi:10.1002/advs.202518878)
Supplement: Supplementary file 1 — Supporting File 1: advs73518‐sup‐0001‐SuppMat.pdf. [file ADVS-13-e18878-s002.pdf]

Supporting Information

MELEGROS: Monolithic Elephant-inspired Gripper with Optical Sensors

Petr Trunin† *Diana Cafiso*† *Anderson Brazil Nardin*† *Trevor Exley** *Lucia Beccai**

Workflow

Monolithic systems that integrate sensing and actuation into the fabrication process require specific workflows. As soft robotic simulation techniques advance, iterative design can be reinforced within the SOFA framework, as presented in Figure S1. The process starts with the design: an actuation unit is printed (in this case, a half-embedded actuator) to assess printability and understand the kinematics during actuation. In parallel, the chosen lattice is printed to inform the homogenized envelope stiffness in the simulation. From here, the design of the gripper is simulated and observed to inform the sensor design. Depending on the sensor geometry, tactile and proprioceptive regions of interest are initially estimated. Representative positions of these regions are tracked during full range of movement (from -50 kPa to 50 kPa). If these observed trajectories produce (in the embedded sensors) the required bending amplitude so that a sensing response is elicited, then the sensors are physically added to the simulated gripper model. After minimal iterations, the same trajectories are collected to ensure that the addition of sensors does not change mechanical behavior (*e.g.*, change in bending angle $< 5°$), and waveguide behavior is decoupled (*e.g.*, tactile waveguide angle $< 5°$ during finger bending). From here, the design with integrated sensors is printed and tested for characterization and grasping applications. Only 4 different prints are required in the entire process: the actuation unit and lattice are printed first, and after sensor placement, a sensorized actuation unit for characterization and a gripper with integrated sensors are printed. This dramatically reduces multiple trial-and-error iterations. Closed-loop control is not addressed in this study and will be addressed in future work.

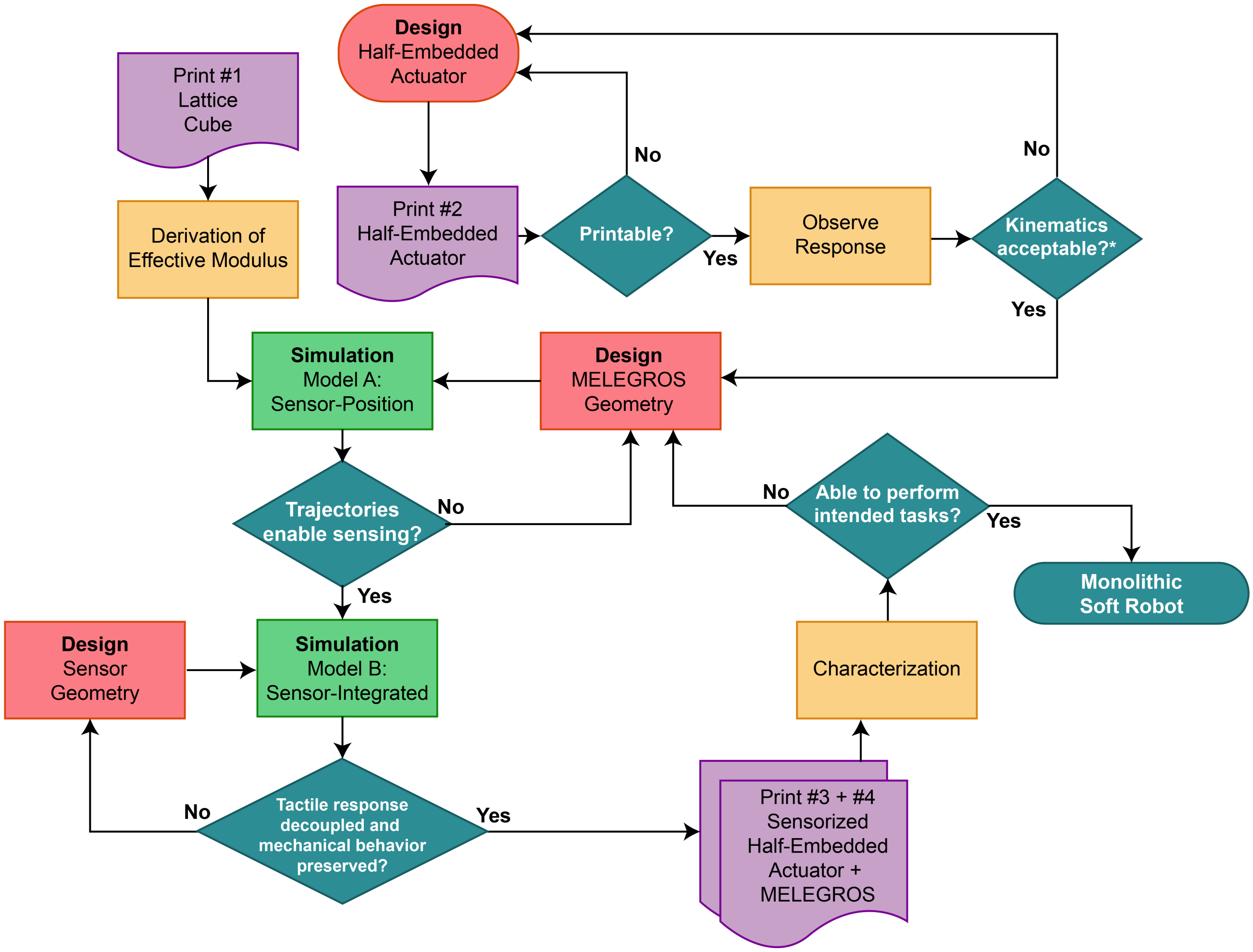

Figure S1: Workflow for MELEGROS development. *Observed kinematics should show that bending is representative to that of a finger to be deemed acceptable.

Lattice Pattern

The IWP-type (Schoen I-graph Wrapped Package) TPMS (Triply Periodic Minimal Surface) lattice is defined by the following equation [1]:

$$F(x, y, z) = 2(\cos(2\pi\frac{x}{L})\cos(2\pi\frac{y}{L}) + \cos(2\pi\frac{y}{L})\cos(2\pi\frac{z}{L}) + \cos(2\pi\frac{z}{L})\cos(2\pi\frac{x}{L})) - (\cos(4\pi\frac{x}{L}) + cos(4\pi\frac{y}{L}) + \cos(4\pi\frac{z}{L})) = t \quad \text{(S1)}$$

in which L is the unit cell size and t is the isovalue. In this study, the lattice was generated by 4D_Additive Manufacturing Software Suite (Coretechnologie, Germany) with direct control over the unit cell size and minimum thickness of the struts. Cyclic compression tests were conducted on a cube of the lattice structure to inform the mechanical properties of the simulation (Figure S2).

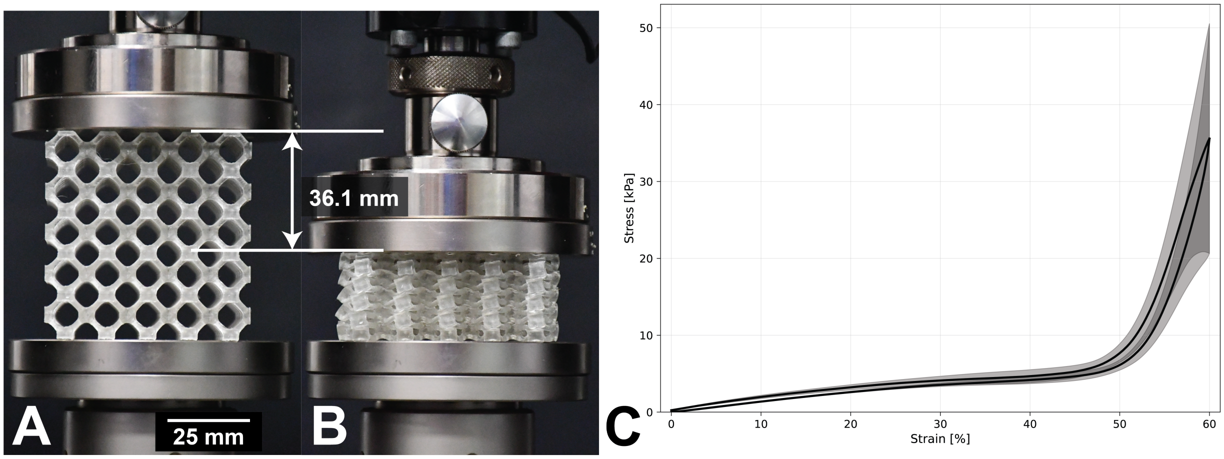

Figure S2: Lattice characterization test. Lattice in (A) undeformed and (B) deformed state. (C) Resulting stess-strain curve. Shaded areas represent ±1 standard deviation (N = 5)

Actuator Design and Scalability

Bladder-like actuators were selected to target both compression and elongation in MELEGROS, under negative and positive pressure, respectively, with negligible radial expansion (Figure S3).

The dimensions of these actuators are directly related to the connections to the IWP-type TPMS lattice. Thus, it is important to design the bladder diameter and length accordingly. For example, given a unit cell size of L, the separation between bladders should equal L, and the diameter of each bladder should equal $2L$ (Figure S4).

Preliminary scaling studies confirmed that both lattice and actuators can be uniformly enlarged or reduced without altering the fabrication workflow, however smaller chambers prove more difficult to remove uncured material during post processing (Figure S5). The strain-limiting behavior was achieved by embedding actuators halfway into the lattice (Figure S6): radial expansion was constrained while bending was directed along the gripper's axis. In addition, the lattice provided support during the 3D-printing process, ensuring stable fabrication of the thin-walled bladders and complex geometries. Since the lattice and actuator size are dependent on each other, the smaller the actuator also significantly increases the amount of required material for the lattice.

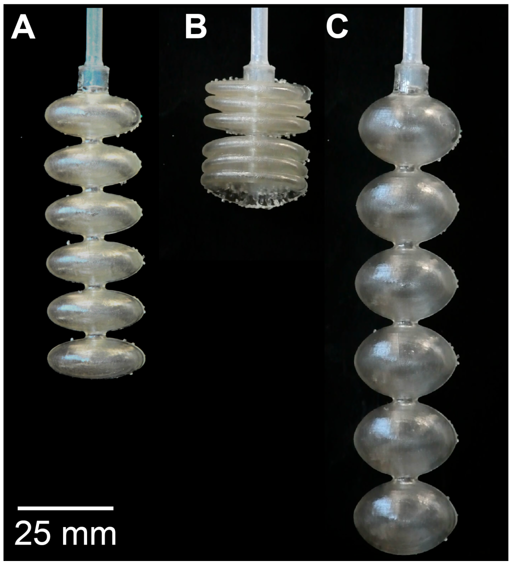

Figure S3: Bladder-like actuator (d = 25 mm) (A) at rest, (B) vacuum (−50 kPa), and (C) pressurized (50 kPa).

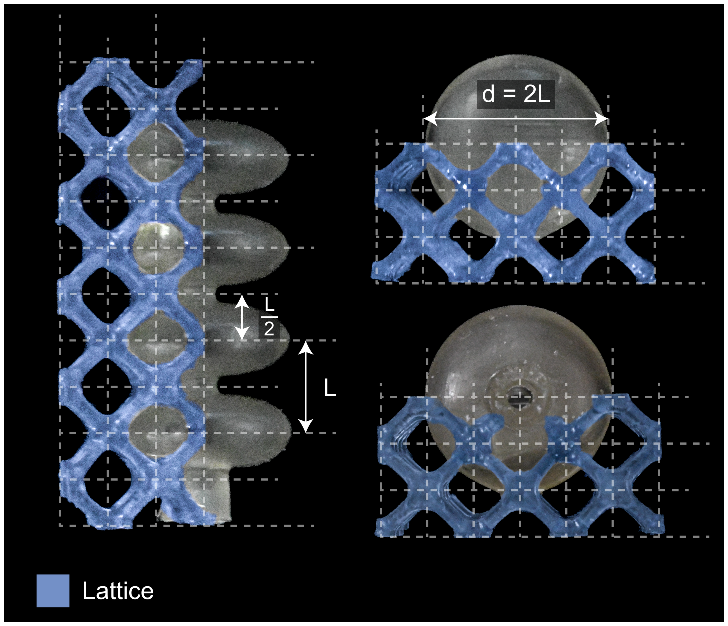

Figure S4: Spacing of the IWP-type TPMS lattice with respect to the bladder when in half-embedded configuration. Bladder dimensions such as d, diameter, are dependent on L, unit cell size.

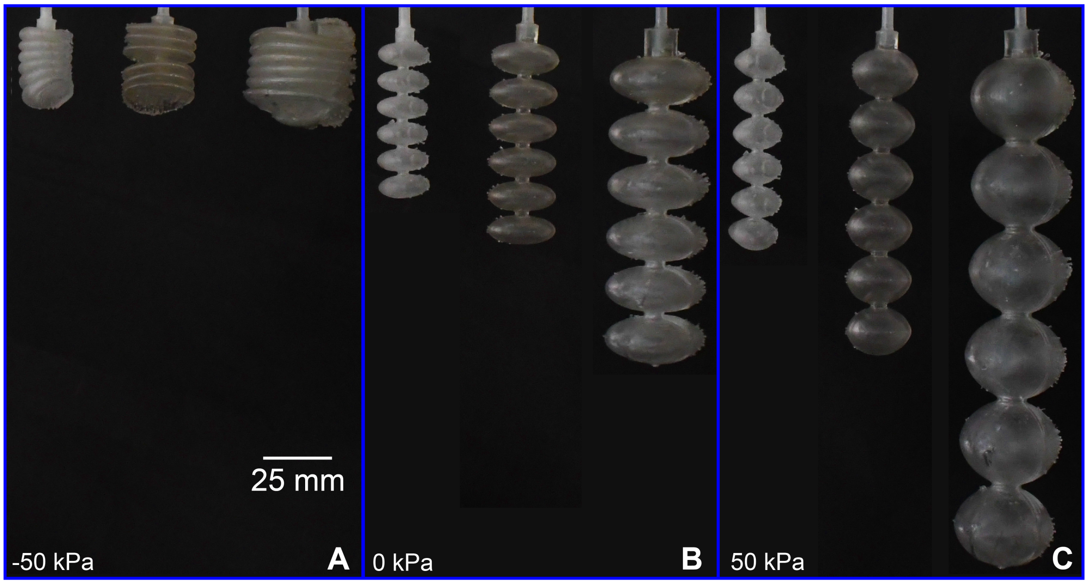

Figure S5: The bladder-like actuators (d = 25 mm) scaled 0.75, 1.0, and 1.25 in the (A) compressed, (B) resting, and (C) actuated states.

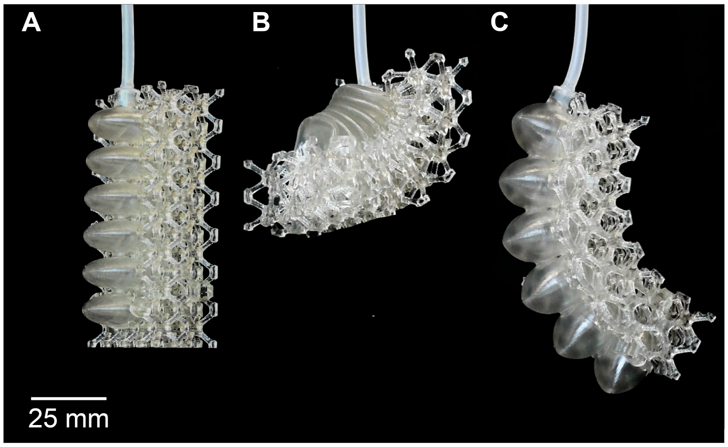

Figure S6: Half-embedded bladder-like actuator (A) at rest, (B) vacuum (−50 kPa), and (C) pressurized (50 kPa).

Gripper Design

The final design of MELEGROS is shown in Figure S7 without sensor placements. The transparent envelope (bulk volume) is input to 4D_Additive Manufacturing Software Suite to generate the lattice body. The base and finger actuators are located with respect to the lattice as Figure S4 depicts. The fingers are designed asymmetrically, with varying geometry (width, profile, thickness) and number of actuator chambers detailed in Figure S7B,C. The dorsal actuator spans the entire finger length and drives 'active' movement, while the ventral actuator covers only a fraction of the finger, leaving the distal part 'passive'. This asymmetry produces more behaviorally bioinspired motions, such as scooping.

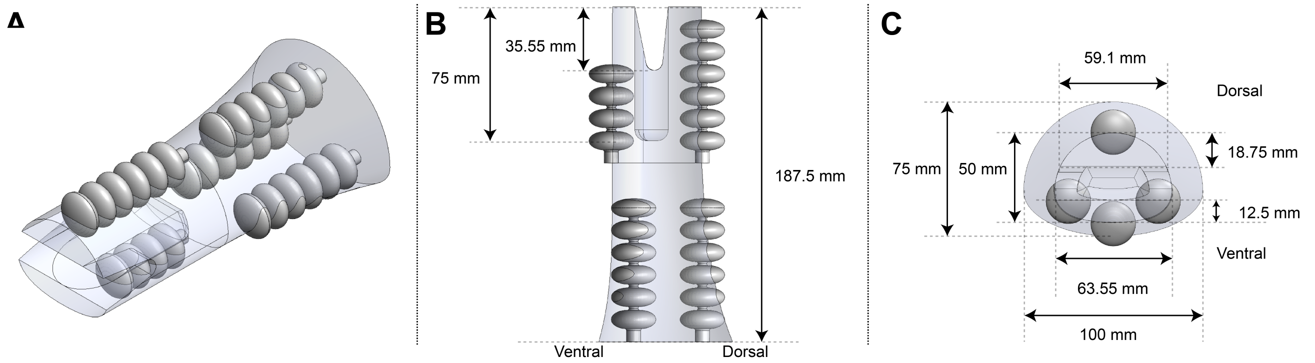

Figure S7: Design of MELEGROS (A) isometric view, (B) frontal view, and (C) top view, with dimensions. The envelope used to generate the lattice is shown in transparency.

Sensor Design

FEM simulations were performed in COMSOL Multiphysics® (COMSOL Inc., Sweden) using a 2D approximation to reduce computational cost. The Ray Optics and Solid Mechanics interfaces were coupled to capture deformation-dependent optical loss. The waveguide was modeled as a rectangular domain with an emitter at one end and a Ray Counter at the opposite end.

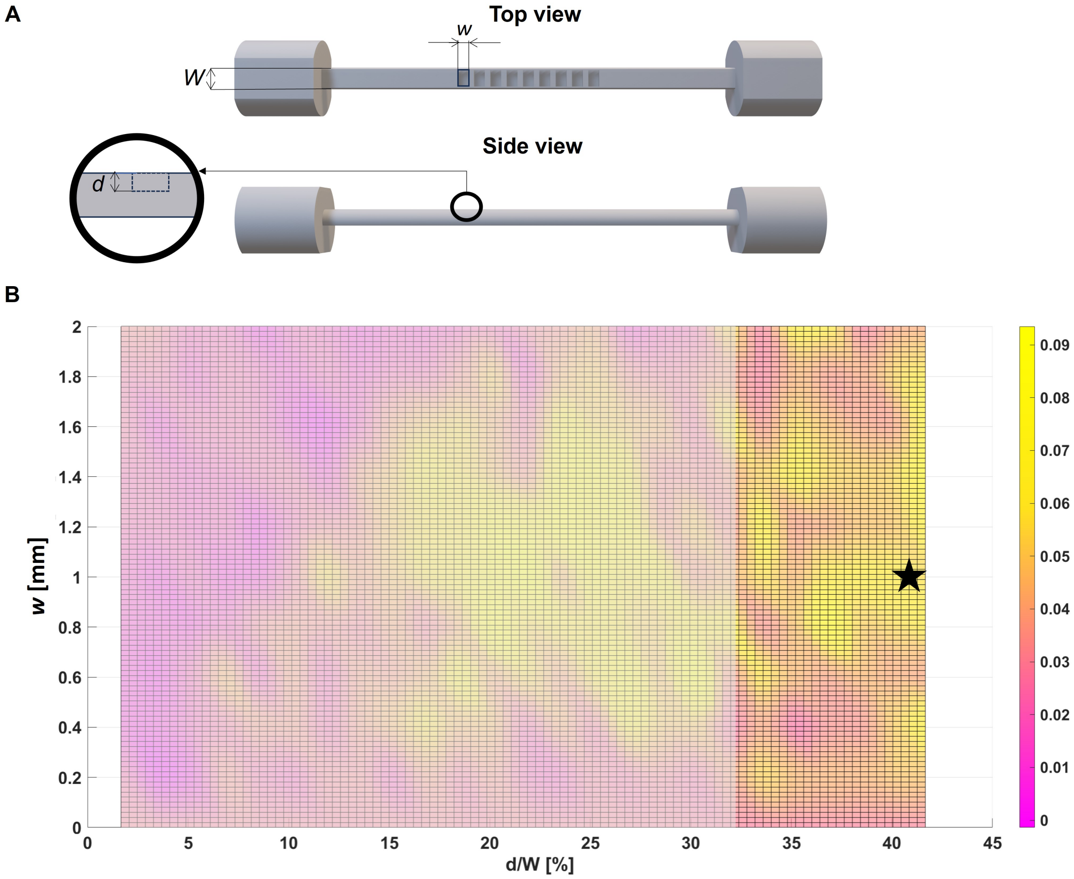

Figure S8: (A) Waveguides design choice based on the printability and (B) simulation in COMSOL with scale bar for Goodness Coefficient..

To identify geometries that maximize sensitivity while preserving linearity, we defined a Goodness Coefficient (GC) as the ratio of the optical intensity drop (ΔI) to the RMSE of a linear regression. The bigger the GC, the better the sensing performance is expected. Two main parameters were used in the simulation: the width of the superficial pattern, w, and the ratio between the depth of the pattern, d, and the width of the waveguide, W. There were 25 parametric steps for d and 20 for w. Furthermore, the size of the electrical components (1 mm) was considered when defining the depth of the pattern (the bottom of the well must not coincide with the photoemitter by default). The simulation was explained further in Trunin et al. [2](Figure S8).

Both proprioception and tactile sensors are designed and integrated in the lattice structure with superficial wells on the side of planned deformation(bending). The tactile sensor bends due to external stimuli, while the proprioceptive sensor bends due to the structural bend (Figure S9).

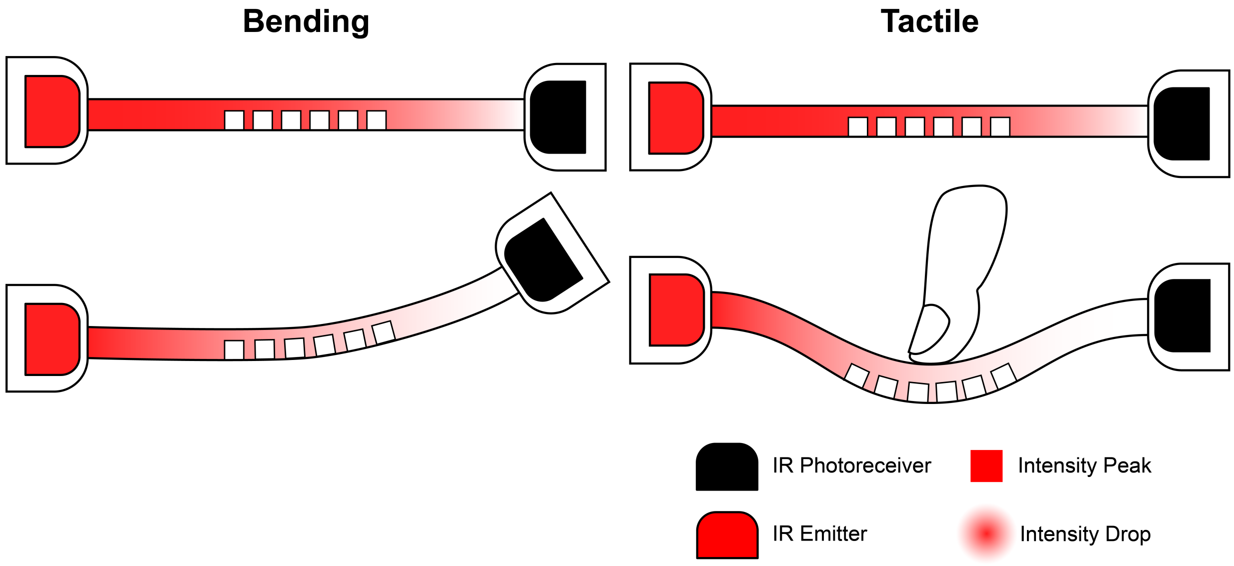

Figure S9: Working principle of bending and tactile sensors. Light intensity drops after deformation in the planned direction.

Cyclic test

Sensor data were recorded during cyclic tests of the actuator. For the blocking force test, 100 cycles of positive (50 kPa) pressure were applied. For the free-bending test, 100 cycles of positive (50 kPa) and negative (-20 kPa) pressures were applied (Figure S10).

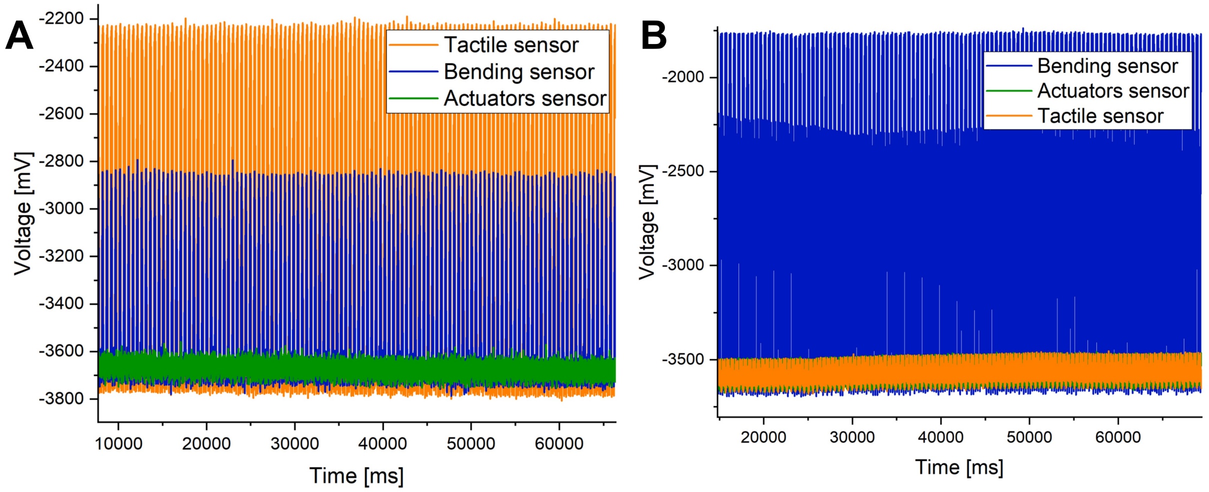

Figure S10: Sensor data from the tactile, bending, and actuator sensors during the cyclic test of the bladder-like actuator from (A) blocking force and (B) free bending tests.

Sensor data were collected during cyclic actuation tests of the MELEGROS system. The gripper was repeatedly opened and closed for 20 minutes to evaluate the stability of the sensors' baseline signals. The test duration was selected based on safety considerations associated with the operation of the pneumatic pumps (Figure S11).

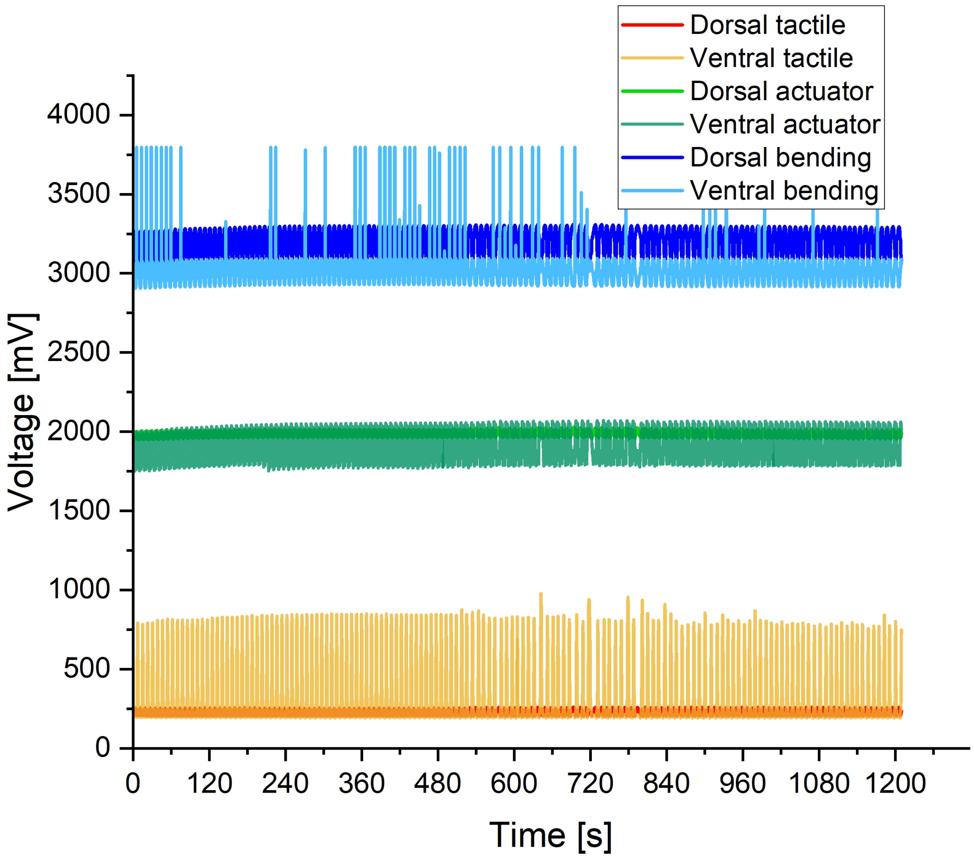

Figure S11: Sensor data of all 6 sensors of MELEGROS during cyclical opening and closing of the gripper for 20 minutes.

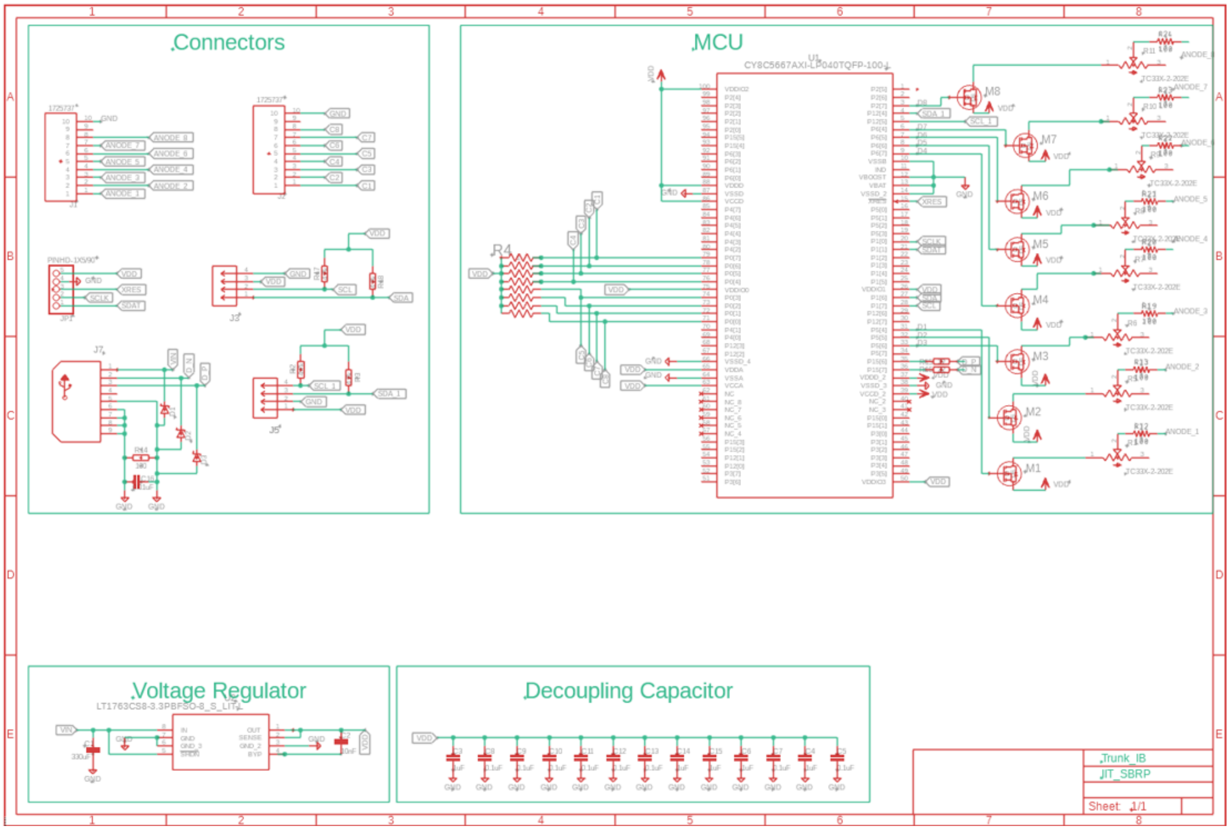

Figure S12: The schematics of the custom PCB used to operate and read out the sensors.

Simulation Framework

The mechanical properties assigned to the simulated gripper follow directly from the homogenization of the lattice material, derived from compression tests of cubic representative samples (Figure S2). The experimentally measured nonlinear response was linearized up to 40% strain and modeled with an effective Young's modulus of approximately 12 kPa, which was applied uniformly to the bulk lattice envelope. The simulations assume quasi-static behavior, linear elasticity within the 40% strain regime, and a homogenized material response for the lattice envelope, while neglecting viscoelastic and fluid–structure coupling effects beyond the applied cavity pressures. In assigning this homogenized material model, we assume that the tested cubic lattice sample behaves as a representative volume element of the gripper's lattice, so that its effective modulus can be applied uniformly to the bulk lattice envelope, neglecting local geometrical defects and spatial variations in stiffness. Pneumatic membranes were treated as deformable subregions mapped onto this volume, while optical waveguides were defined as stiffer elastic domains. All portions (lattice volume, membranes, cavities, and, when applicable, the optical waveguides) were meshed using a fully scripted Gmsh workflow that imported the STEP files, removed duplicates, applied Boolean fragmentation to resolve overlapping volumes, and generated a tetrahedral mesh using the Delaunay 3D algorithm. To ensure reproducible mesh density, characteristic element lengths were controlled through a global mesh size factor (0.5), with linear (first-order) tetrahedral elements enforced. Additional smoothing (one iteration) ensured stable element quality across thin membranes and narrow waveguide regions. These discretization choices assume that first-order tetrahedral elements and the selected mesh density provide sufficient accuracy to capture the overall deformation patterns while keeping the computational cost tractable. The resulting component meshes were then merged into a single unstructured grid by node-proximity consolidation, ensuring consistent connectivity across material interfaces. Actuation was driven by time-varying pressure functions applied to the cavity surfaces, with dedicated scripts interpolating pressure profiles, applying boundary conditions at each time step, and recording the nodal trajectories in predefined regions of interest. This modeling framework defines the procedures used in the subsequent design iterations, including the evaluation of sensing feasibility and the influence of embedded waveguides on gripper mechanics.

To evaluate actuator kinematics and guide sensing, two simulation cases were implemented in SOFA: the *Sensor-Position Model* (Model A), in which only candidate sensor locations are indicated, and the *Sensor-Integrated Model* (Model B), in which the sensor bodies are also present and therefore contribute mechanically.

Figure S13 summarizes Model A. Figure S13A shows the initial configuration with candidate sensor locations highlighted. Figure S13B–E illustrate finger-specific actuation modes: **open1** and **close1** for the dorsal finger, and **open2** and **close2** for the ventral finger.

Figure S14 reports Model B, where sensors are embedded in the mechanics. Figure S14A shows the initial state; Figure S14B depicts the **grasp** sequence. Figure S14C–D illustrate **elongate** and **contract** of the proximal chambers. The sensing layout comprises four sensor regions, namely *Dorsal actuator*, *Dorsal bending*, *Ventral actuator*, and *Ventral bending*.

Figure S14E–J present additional finger actuation modes: **open** and **close** involve simultaneous motion of both fingers, whereas **open1/close1** correspond to the dorsal finger and **open2/close2** to the ventral finger. The integrated sensing layout comprises six sensors, namely *Dorsal actuator*, *Dorsal bending*, *Dorsal tactile*, *Ventral actuator*, *Ventral bending*, and *Ventral tactile*.

To reproduce the simulations, it is provided two reference algorithms. The first algorithm (Algorithm 1) focuses on creating the simulation environment, defining the soft arm's physical parameters, importing the meshed subdomains (lattice/actuator volume, membranes, cavities, and, when applicable, sensor inclusions), and linking all models, ROIs, collision layers, and visualization. The second algorithm (Algorithm 2) provides the control logic that applies time-varying pressure inputs according to predefined regimes (the different modes of operation), while tracking motion via monitored points, logging reaction

forces in a region of interest, and enabling manual teleoperation.

Algorithm 1 Scene Construction

Initialize SOFA core (animation loop, solvers, collision pipeline, visualization); set dt and gravity
Add environment plane (mesh $\rightarrow$ topology $\rightarrow$ collision $\rightarrow$ visual)
Create rigid target (pose, mass/inertia); add collision shell and visual mapping
Define list *Actuators* with pose parameters (rotation, translation)
for each actuator $\in$ *Actuators* **do**
  Soft body (tet mesh)
    Load merged tetrahedral mesh; create topology and mechanical state
    Fix base using *BoxROI* + *RestShapeSprings*; define distal ROI for force readout
    Monitors:
  for $j = 1$ **to** 6 **do**
      Attach *Monitor* to a triplet of node indices; enable position export
  end for
  Membrane subdomains
    Gather STL files: `membrane*.stl`
  for each membrane file **do**
      Build *MeshROI* on host tets; add dedicated FEM block
  end for
  Sensor subdomains (Model B)
    Gather STL files: `sensor*.stl`
  for each sensor file **do**
      Build *MeshROI* on host tets; add elastic waveguide block
  end for
  Pneumatic cavities
    Gather STL files: `cavity*.stl`
  for each cavity file **do**
      Load surface; add *SurfacePressureConstraint* (pressure value-type) with barycentric mapping
  end for
  Collision layer
    Load outer surface; add triangle/line/point collision models and mapping
  Visualization
    Overlay translucent lattice; for $j = 1, \ldots, 6$ overlay colored sensor visuals; map to tets
end for
Attach controller (options: save on/off, automatic on/off, selected input regime); **return** root node

Algorithm 2 Pressure Controller

Discover cavities in the scene; cache handles to their *SurfacePressureConstraint*s
if automatic mode **then**
  Load CSV profile for selected regime (open, close, open1, open2, close1, close2, elongate, contract, grasp)
  Build per-cavity sequences; set pre/post holds; compute total duration
end if
if saving enabled **then**
  Open output CSV
end if
while simulation running **do**
  if automatic window active **then**
    for each cavity **do**
      Set pressure value from time-indexed sequence
    end for
  end if
  Read back pressures; sample forces in ROI; compute (F_x, F_y, F_z) and selected distances
  if saving enabled and past pre-hold **then**
    Append timestamp and pressures to CSV
  end if
  if automatic mode and time > total duration **then**
    Stop simulation
  end if
  Teleoperation (manual mode): number keys → adjust cavity pressures; arrows → translate rest pose; "."/"/" → rotate rest pose
end while

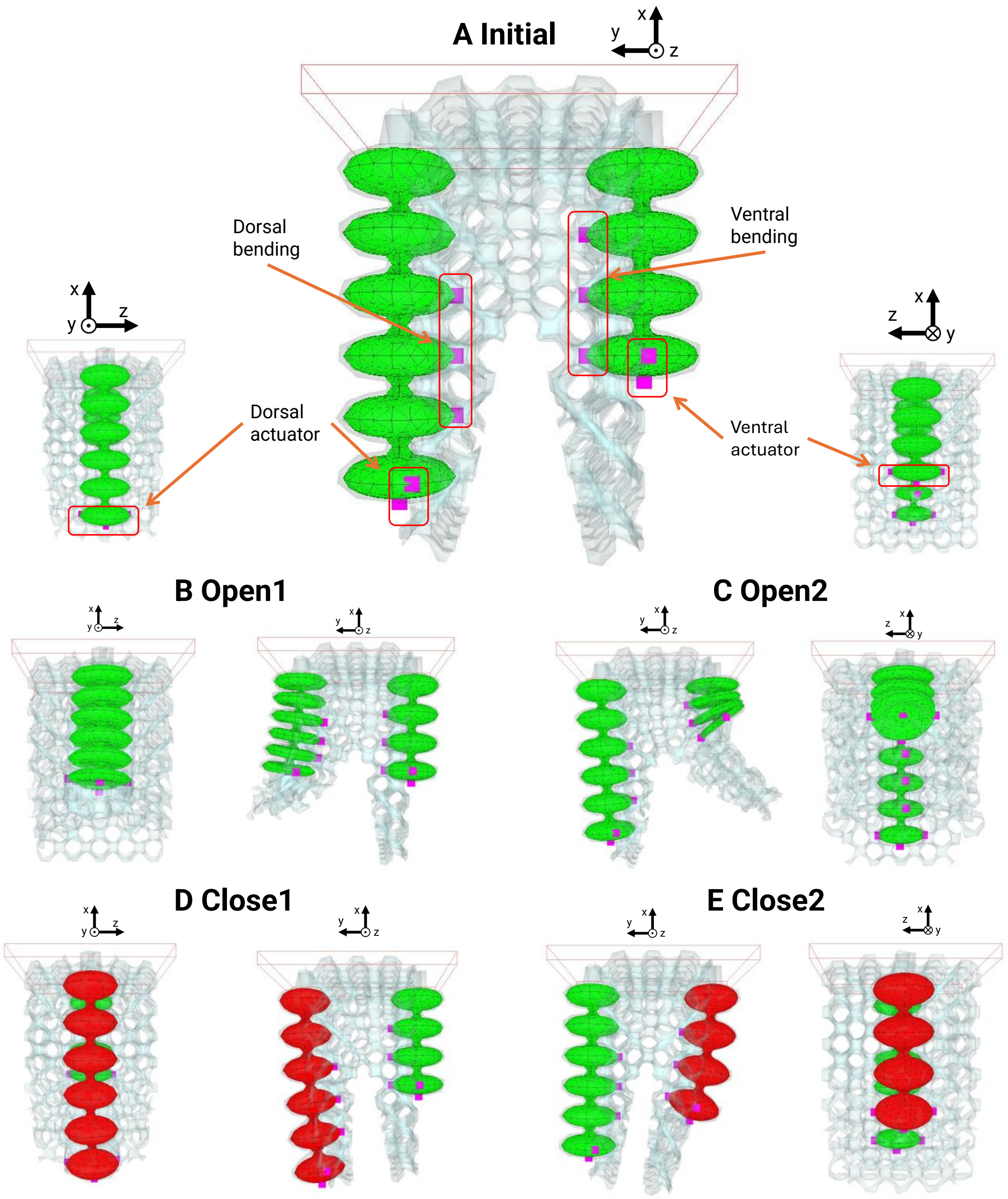

Figure S13: SOFA simulation setup for the *Sensor-Position Model* (Model A). A: Initial configuration with candidate sensor locations highlighted. B: Finger **open1** (dorsal finger). C: Finger **open2** (ventral finger). D: Finger **close1** (dorsal finger). E: Finger **close2** (ventral finger). Green indicates zero to negative pressure, red indicates positive pressure, and pink marks the sensor positions of interest.

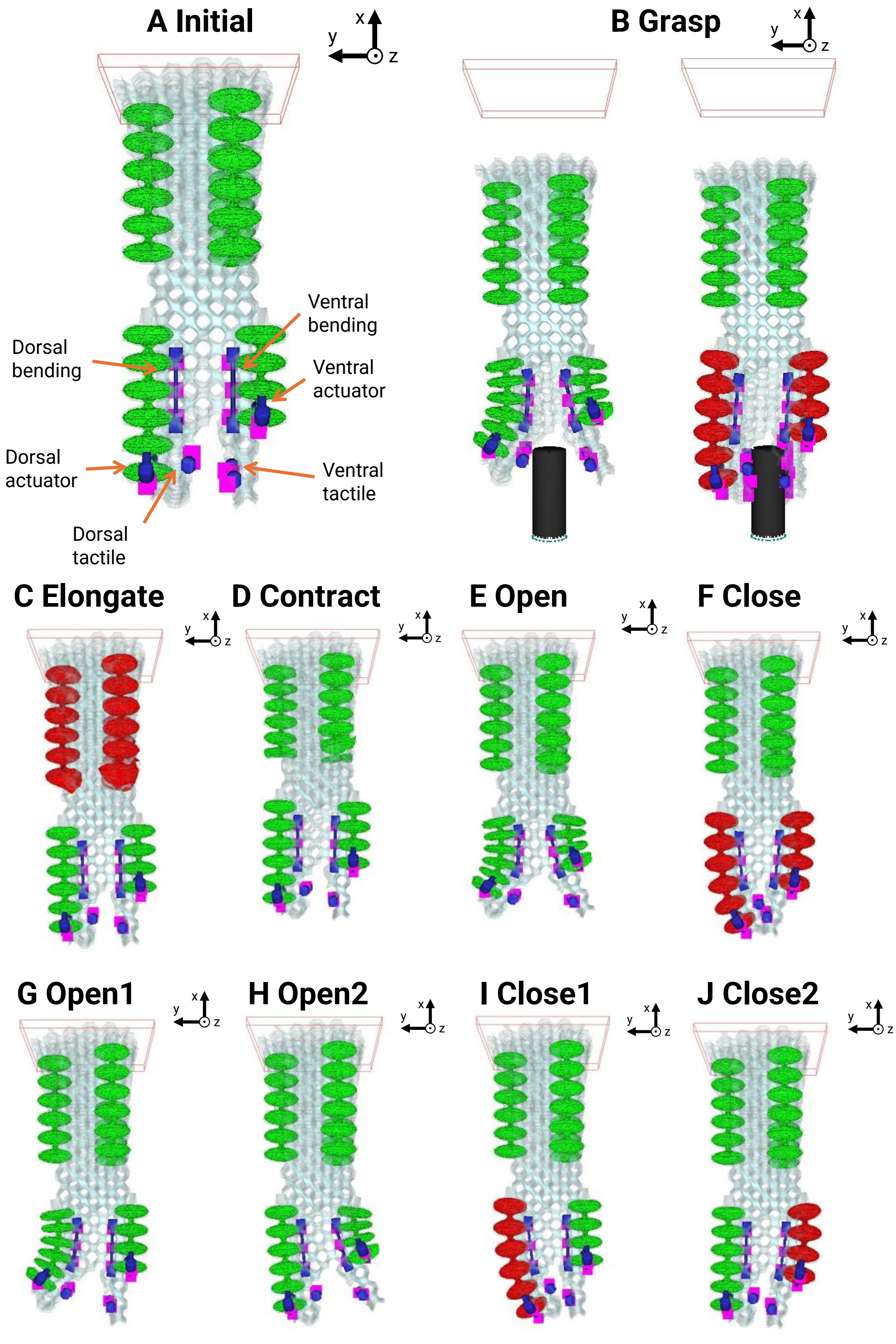

Figure S14: SOFA simulation setup for the *Sensor-Integrated Model* (Model B). A: Initial configuration with integrated sensors. B: **Grasp**. C: **Elongate** proximal chambers. D: **Contract** proximal chambers. E: **Open** (both fingers simultaneously). F: **Close** (both fingers simultaneously). G: **open1** (dorsal finger). H: **open2** (ventral finger). I: **close1** (dorsal finger). J: **close2** (ventral finger). Green indicates zero to negative pressure, red indicates positive pressure, pink marks monitored points, and blue marks the sensors that are integrated.

The 2D projections of the sensor-point trajectories for Model A are provided under ventral-finger modes. These plots complement Figure S13 by making the spatiotemporal paths explicit: **open2** in Figure S15 and **close2** in Figure S16.

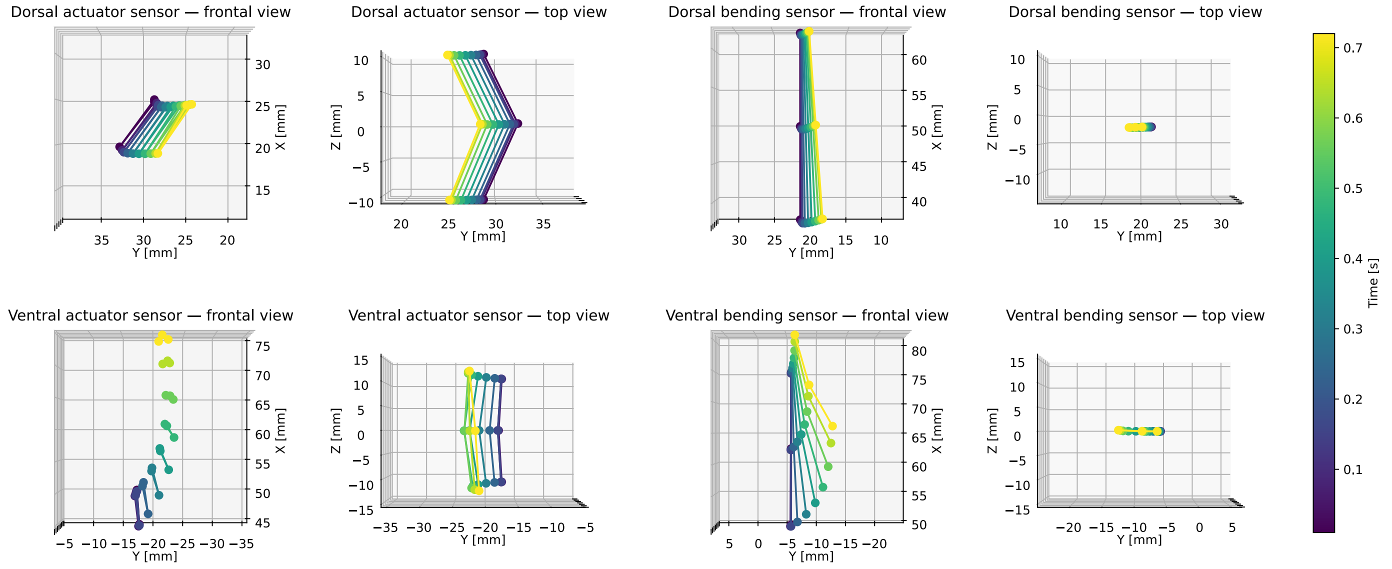

Figure S15: 2D projections of sensor trajectories for the *Sensor-Position Model* (Model A). Finger **open2**.

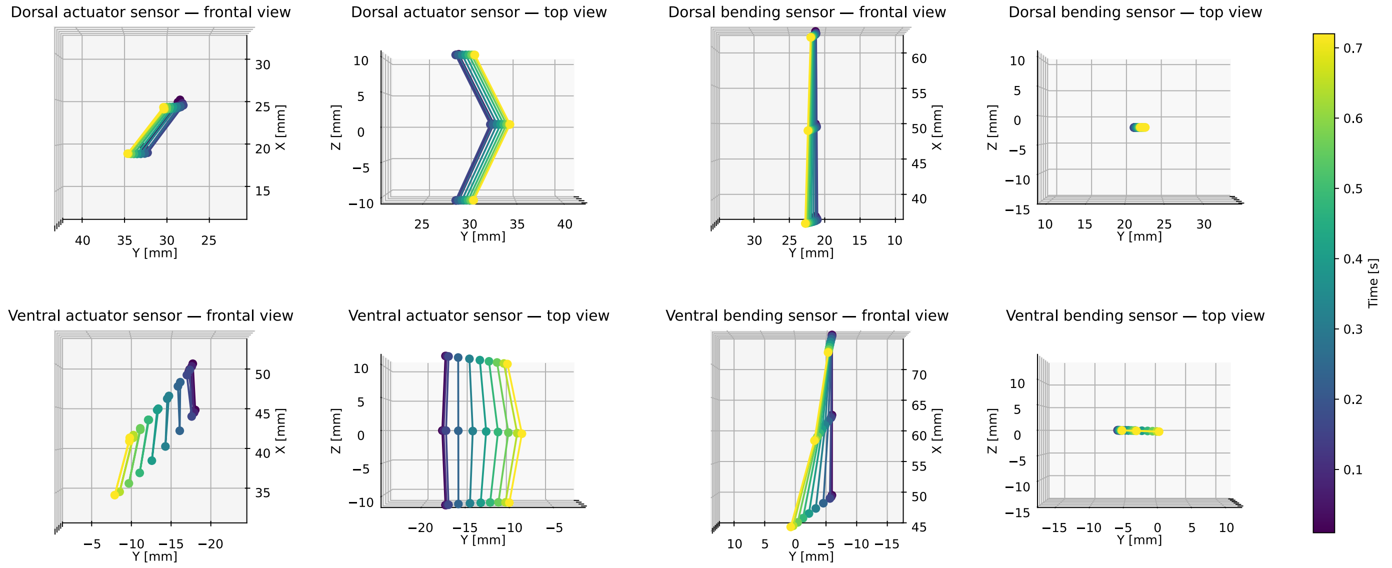

Figure S16: 2D projections of sensor trajectories for the *Sensor-Position Model* (Model A). Finger **close2**.

Then, it is reported the corresponding projections for Model B. These results visualize the effect of the integrated sensors on the kinematics across the finger-simultaneous modes: **open** (Figure S17) and **close** (Figure S18).

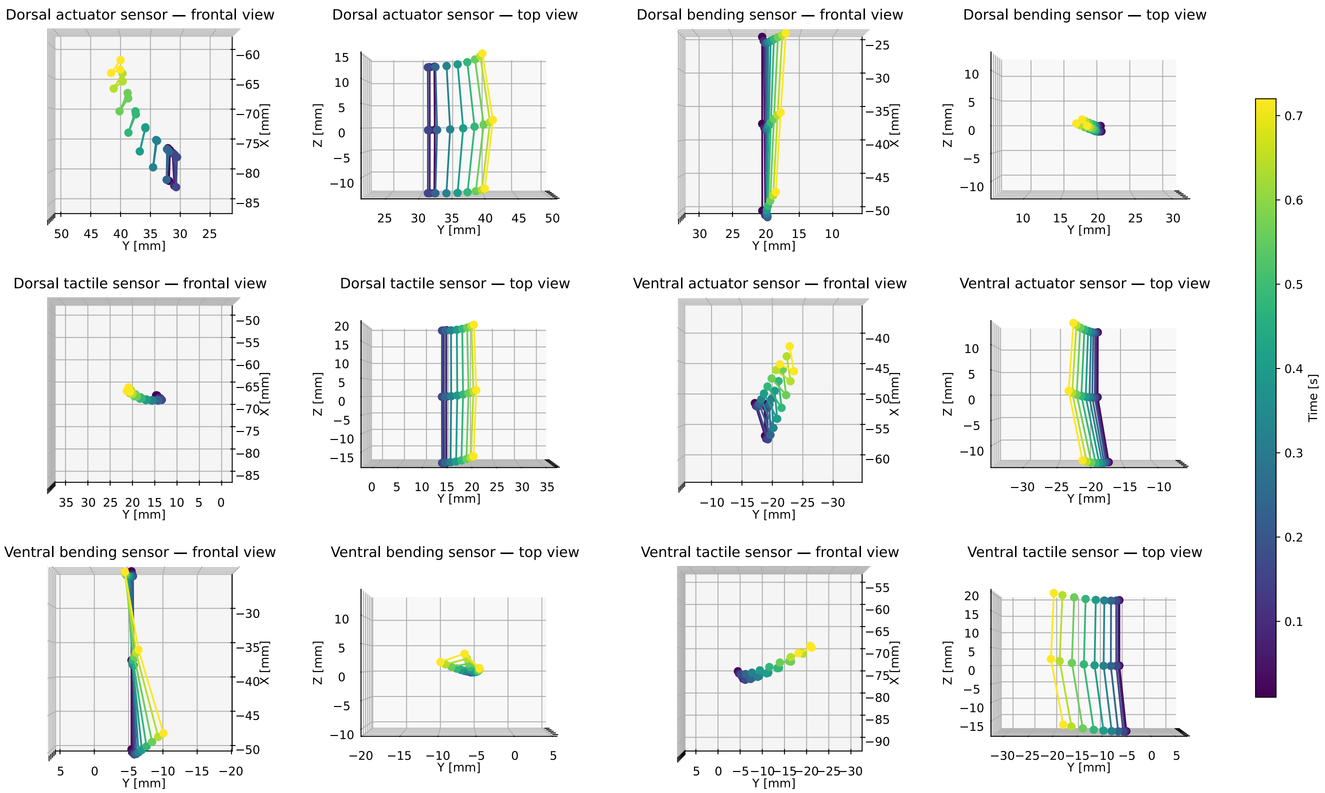

Figure S17: 2D projections of sensor trajectories for the *Sensor-Integrated Model* (Model B). Fingers **open**.

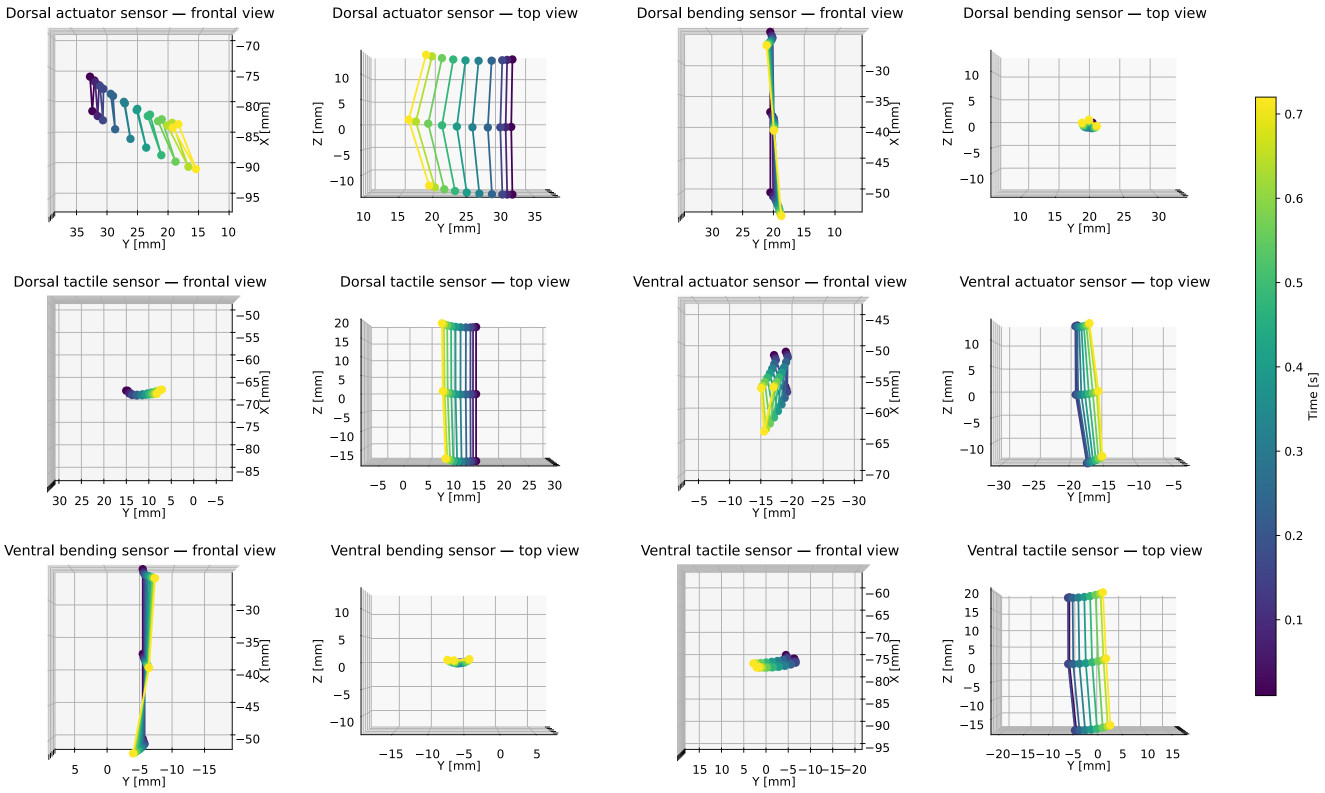

Figure S18: 2D projections of sensor trajectories for the *Sensor-Integrated Model* (Model B). Fingers **close**.

In Model A (Figure S15 and S16), the frontal-view trajectories provide clear evidence of the suitability of the site. The **open2** and **close2** modes correspond to ventral-finger actuation, where the finger is driven into opening and closing, respectively. In the ventral actuator sensor during the **open2** mode, the trajectory extends over roughly 20–30 mm, capturing large-scale motion of the finger. Under the **close2** mode, the same site still spans more than 15 mm, confirming its sensitivity across bi-directional actuation regimes. By comparison, the ventral bending sensor shows trajectories confined to narrow bands of about 5–10 mm in both modes, reflecting localized but consistent deformation. The nature of these responses supports the adoption of both sensor types for integration in Model B. In Model B (Figure S17 and S18), the **open** and **close** modes correspond to the simultaneous actuation of both fingers. The actuator sensors trace excursions of about 20–25 mm in the **open** mode and remain around 15 mm in the **close** mode, showing that the integration of sensor bodies preserves their ability to capture large-scale motion across bi-directional actuation regimes. Bending sensors follow more confined trajectories in the 5–10 mm range, consistent with localized deformation signals identified in Model A. Dorsal and ventral sites remain well separated, with smooth, distinct traces that reflect complementary roles in encoding overall motion and local bending.

Figure S19 illustrates a schematic of the modeled MELEGROS, highlighting the distinct components considered in the simulation process. The homogenized outer envelope is shown in green, the internal lattice structure in blue, the actuator membranes in red, the pneumatic cavities in magenta, and the embedded sensors in orange.

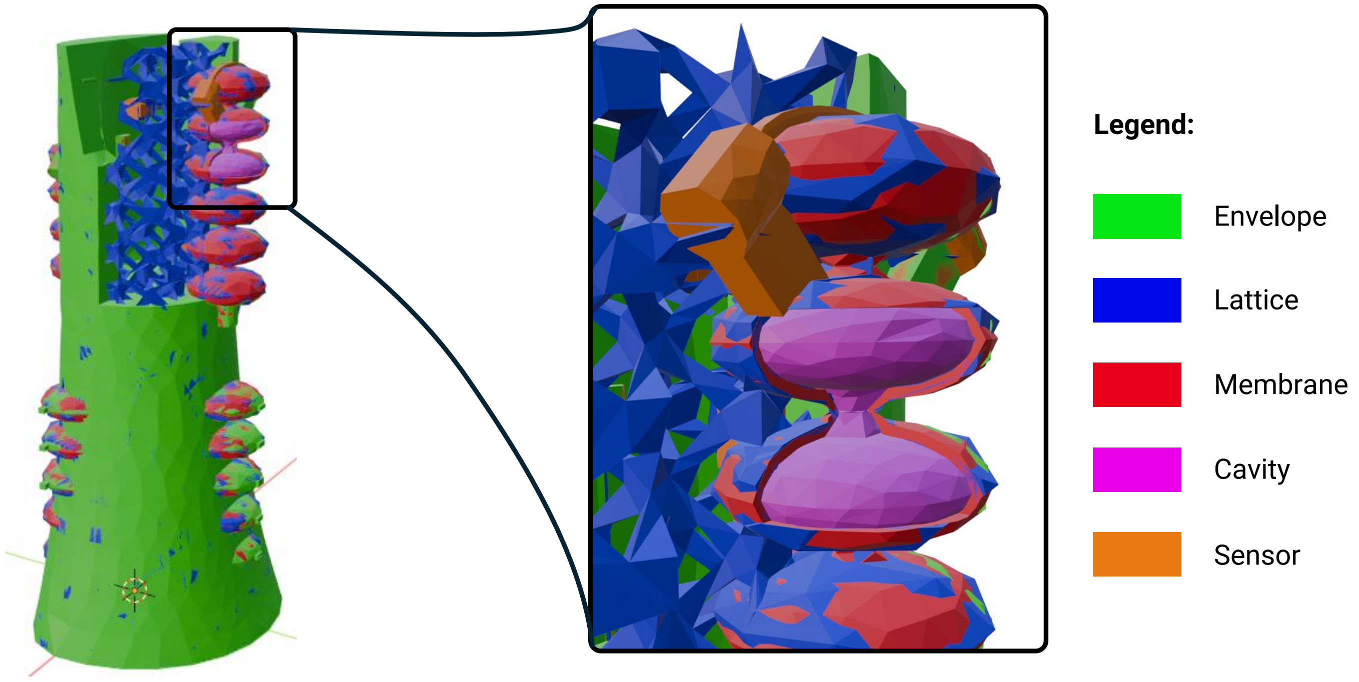

Figure S19: Broken-out section of the soft manipulator highlighting the simulation-relevant regions.

The angular response of integrated sensors was further analyzed for Model B. In particular, the evolution of the angle with respect to the applied chamber pressure was computed for Dorsal and Ventral tactile sensors. The resulting curves are reported in Figure S20.

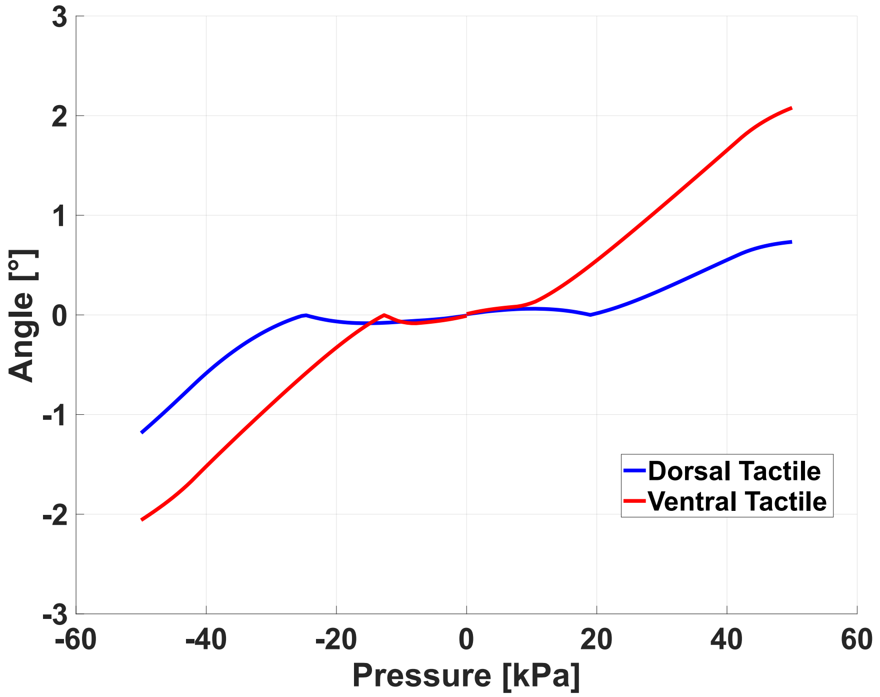

Figure S20: Angle–pressure relationships for Model B obtained from Dorsal tactile sensor (blue) and Ventral tactile sensor (red). The distinct responses illustrate the influence of sensor positioning on measured angular variations.

Additional Application Information

The objects that were grasped by MELEGROS in Video S3 are shown in Figure S21. All objects were printed with ABS with a desktop 3D printer (Ultimaker S7, Netherlands).

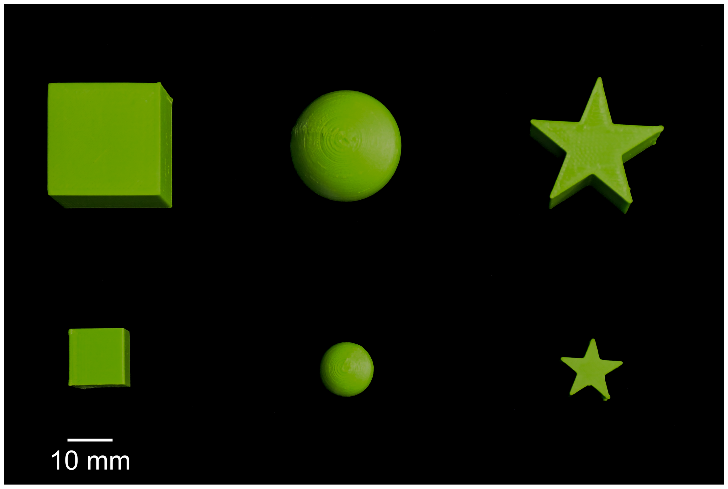

Figure S21: Cube, sphere, and star objects with sizes of (bottom) one unit cell (12.5 mm) and (top) two unit cells (25 mm).

References

[1] C. Zhang, H. Qiao, L. Yang, W. Ouyang, T. He, B. Liu, X. Chen, N. Wang, C. Yan, *Composite Structures* **2024**, *327* 117642.

[2] P. Trunin, D. Cafiso, L. Beccai, *Additive Manufacturing* **2025**, *100* 104687.
